# Trends in the Prevalence and Factors Associated with Undernutrition in Tunisian Children Aged 0–23 Months

**DOI:** 10.3390/nu16223893

**Published:** 2024-11-14

**Authors:** Nagwa Farag Elmighrabi, Catharine A. K. Fleming, Kingsley E. Agho

**Affiliations:** 1 Campbelltown Campus, School of Health Sciences, Western Sydney University, Sydney, NSW 2560, Australiak.agho@westernsydney.edu.au (K.E.A.); 2 Organization of People of Determination and Sustainable Development, Benghazi 18251, Libya; 3 Department of Nutrition, Faculty of Public Health, University of Benghazi, Benghazi 16063, Libya; 4 Translational Health Research Institute (THRI), School of Medicine, Western Sydney University, Penrith, NSW 2750, Australia; 5 Faculty of Health Sciences, University of Johannesburg, Johannesburg 2094, South Africa

**Keywords:** stunting, wasting, underweight, under 2 years, undernourished

## Abstract

Background: In recent years, undernutrition has remained a significant public health issue in Tunisia, increasing the risk of illness and mortality in young children. Therefore, this study aims to analyse the prevalence and factors contributing to undernutrition among Tunisian children aged 0–23 months. Methods: The study included 3265 children aged 0–23 months from the 2011–2023 Tunisia Multiple Indicator Cluster Surveys (MICS). Trends and logistic regression analyses were used to determine the prevalence and predictors of undernutrition. Results: The prevalence of stunting, wasting, and underweight in infants and children aged 0–23 months has increased by 3.3%, 0.5%, and 2.1%, respectively. Stunting and underweight were more common among infants aged 0–5 months (11.8% for stunting, 8.9% for underweight, *p* < 0.01), and first-time mothers (8.3% for stunting, 4.1% for underweight, *p* < 0.01). In 2023, compared to 2011, the odds of stunting, wasting, and being underweight had increased by 22%, 16%, and 70%, respectively. Infants aged 0–5 months had higher odds of undernutrition in all three indices. Children of obese or overweight mothers, and those who started breastfeeding late, were more likely to be stunted. Boys had significantly higher odds of wasting and underweight. Children with low birth weight, and duration of breastfeeding > 12 months, had significantly higher odds of being underweight. Conclusions: This study shows that infants aged 0–5 months, first-time mothers, boys, and children from poor households in Tunisia are at a higher risk of undernutrition. To address the growing issue of undernutrition in Tunisian children, enhancing maternal and child health and nutrition services, improving parental education, and implementing community-based programs that provide breastfeeding and nutritional education to infants born to new mothers and mothers with high/low BMI is recommended.

## 1. Introduction

The most rapid growth period occurs during the first 2 years of life [1]. During this critical period, adequate nutrition is essential to prevent stunting and undernutrition, which can have long-term effects on cognitive development, educational achievement, and future economic productivity [1,2]. Adequate nutrition during this crucial period strengthens an infant’s immune system and reduces the risk of chronic diseases, such as obesity and diabetes, later in life [1]. Undernutrition in children is a significant public health issue in many low- to middle-income countries (LMIC). Undernutrition is the underlying cause of 35% of child deaths and contributes to 11% of the global disease burden [3]. Undernutrition is a key predictor of poor growth and morbidity in infants and young children [1,2]. Along with this, undernutrition can lead to delayed cognitive development and poor school performance in middle childhood and adolescence, ultimately reducing the potential earning capacity of children in the future, thus perpetuating poverty [1]. In addition, undernutrition has a broader impact by reducing the physical and cognitive abilities of the workforce, leading to hindered national development and economic productivity [2]. Tackling undernutrition is not just about public health; it also plays a crucial role in promoting long-term economic growth and social stability [1].

In 2022, it was estimated that about one in five African children were affected by undernutrition, with 31% presenting with stunting [4]. This accounted for approximately 79% of the average number of under-five deaths in the region [5]. North Africa reported the lowest average number of deaths [5]. However, the situation in North Africa has worsened due to factors such as recent political instability, poverty, climate change-induced natural disasters, and the recent pandemic. Challenges such as these can worsen mother and child health issues, placing additional pressure on vulnerable communities by limiting the availability of maternal and child healthcare services, food, and other essential resources, such as clean water and sanitation, which places additional burden on vulnerable communities, making the issues of malnutrition and disease even worse [6,7,8]. The negative impact of undernutrition extends to national economies by reducing productivity and increasing healthcare costs, thereby straining education and health systems and potentially triggering a high rate of malnutrition in the region [6].

Investing in research and interventions aimed at improving maternal and child nutrition, particularly in low- and middle-income countries like Tunisia, has the potential to break the cycle of poverty [1,9]. Targeted investment for research programs not only enhances the well-being of mothers and children but can also promote economic and social development, ultimately contributing to a more equitable and prosperous society. Focusing on nutrition during the first 1000 days (0–2 years) establishes a strong foundation for sustained development and economic stability [1,2,10].

Previous studies on undernutrition in Tunisia are limited. For example, a study which assessed poverty and child health from a population-based cross-sectional perspective revealed that household wealth, education, and location were the main factors influencing a child’s health, including undernutrition [11]. The study focused solely on factors associated with inequality, such as residence, mother’s education, and household wealth index; it did not examine other factors such as dietary practices, and child health, which are directly related to child health. Additionally, the study used a single data source, which limited the scope for comprehensive conclusions. Previous studies on undernutrition in North Africa, which included Tunisia, may not adequately capture the country’s unique context [12,13,14]. As a result, their findings could either overestimate or underestimate Tunisia’s nutritional status, leading to ineffective interventions. This is particularly significant when comparing Tunisia to neighbouring countries with varying rates of undernutrition. Therefore, there is an urgent need for comprehensive research that includes all malnourished children and considers a wide range of factors to develop effective policies.

Therefore, this study aimed to investigate the prevalence, trends, and determinants of undernutrition among Tunisian children aged 0–23 months. The study utilised data from the three most recent nationally representative MICS surveys conducted in Tunisia in 2012, 2018, and 2023.

## 2. Materials and Methods

### 2.1. Study Design and Setting

Data for the current study were obtained from the Tunisia Multiple Indicator Cluster Surveys (MICS) that were conducted in 2011-12, 2017-18, and 2022-23. They are nationally representative surveys, employ standard methods, and are publicly available on the MICS website [15]. Every 5 years, the MICS system in Tunisia is collected on a regular basis [16]. MICS is a global data collection system designed to gather periodic cross-sectional data. Its purpose is to provide internationally comparable information on fundamental health indicators, such as child mortality, maternal health, immunization, nutrition, sanitation, access to clean drinking water, education, and child development.

MICS was schemed in such a way as to provide internationally comparable information on basic health indicators, especially the indicators that are associated with maternal and child health. The survey can target specific regions, although typically the survey is administered across the entire country. To guarantee consistency, the MICS surveys utilise multistage stratified cluster sampling, with defined selection probabilities for each primary unit.

Both the Tunisian MICS 2012 and 2018 were implemented nationwide by the Ministry of Development, Investment, and International Cooperation in Tunisia and the National Institute of Statistics (Statistics Tunisia). Financially, MICS 2012 was supported by the United Nations Children’s Fund (UNICEF), the United Nations Population Fund, and the Swiss Cooperation Office in Tunisia, while MICS 2018 was supported by UNICEF and the National Institute of Statistics (Statistics Tunisia). The Tunisian MICS 2023 was published by the National Institute of Statistics (Statistics Tunisia) and supported by the Ministry of Economy and Planning, the National Institute of Statistics in Tunisia, and UNICEF.

### 2.2. Study Population and Data Collection

All women aged 15–49 years, who were either permanent residents or visitors present in the households on the night before the survey, were deemed eligible for data collection. Information on child health was obtained from the mothers, particularly concerning the youngest child aged less than five years. Only children between 0–23 months were considered in this study. Detailed information about the sampling design and questionnaire may be obtained from the country-specific MICS reports. The questionnaire specifically for women attracted a 91–99% response rate. Our analysis covered a total of 3265 children aged between zero and 23 months, over the three MICS surveys included in the study: 1271 children from the 2012 MICS, 1337 children from the 2018 MICS, and 657 children from the 2023 MICS. The questionnaires used for this study obtained information on various aspects including education level, marital and work status, place of residence, exposure to the media, history of pregnancy, child mortality, breastfeeding and infant feeding practices, such as complementary feeding, childhood immunisations and illnesses, sexual activity, background characteristics of the husband, and awareness and behaviour associated with HIV/AIDS and other sexually transmitted diseases, as well as maternal mortality.

### 2.3. Outcome Variables

The outcome variables utilised for the study included the three indicators of undernutrition, namely, stunting, wasting and underweight. The prevalence of each indicator was calculated with the 2006 WHO growth reference as a basis [17]. The WHO growth standards reference compares the growth of a child to the median of a healthy child within the same age bracket, or reference population [17]. This reference is often expressed in terms of the number of standard deviations (SDs) above or below the median. Stunting was assessed based on the height-for-age (HAZ) Z-score ≤ −2 SD; wasting was estimated using the weight-for-height (WHZ) Z-score ≤ −2 SD of the median, and underweight was evaluated using the weight-for-age (WAZ) Z-score < −2 SD of the median. To evaluate the degree of stunting, wasting and underweight, the dependent variables were divided into two categories: category 0 for those who were not stunted, wasted or underweight (−2SD) and category 1 for those who were stunted, wasted or underweight (−2SD) [17].

### 2.4. Potential Covariates

A past study carried out on 35 low- and middle-income countries [18], as well as other previous studies [19,20,21,22] were used as the basis for the selection of covariate variables. These covariates were categorized into three main factors: enabling factors, including place of residence (urban/rural); underlying factors, including the pooled household wealth index, child age and gender, parents’ age, maternal education and nutritional health, household members, birth order, health care services use, water and sanitation, and access to media; and immediate factors, which included early initiation of breastfeeding, duration of breastfeeding, perceived size of baby, and child illness (diarrhoea, fever, cough and any infection in the last two weeks).

The “Household Wealth index” is a quantitative indication of a household’s financial situation in relation to its expenses and incomes. The Principal Components Analysis (PCA) approach has been used to establish a quantitative representation of family assets. Following the computation of this measure, each lawful household member was awarded a score to establish their ranking in the population. This study classified wealth into five categories: the poorest, poorer, middle, fourth, and highest at the national level. The poorest and poorer households made up the lowest 40% of households, the next 20% were middle-class homes, and the fourth and richest households made up the top 40%.

### 2.5. Data Analysis

Data analysis was performed using the ‘SVY’ commands of Stata version 17.0 (Stata Corp) to adjust for the cluster sampling design and apply appropriate sampling weights to determine counts and percentages for all sociodemographic characteristics for children aged 0–23 months in the 2011–2012, 2017–2018, and 2018–2023 MICS datasets. Survey (svy:tab) tabulation was used to determine the proportion and their 95% confidence intervals of undernutrition (stunting, wasting and underweight) by three time periods (2011–2012, 2017–2018, and 2018–2023). To determine the trends and their 95% CI, the three time periods were classified into three categories. The 2011–2012 period is arbitrarily referred to as Category 1, the 2017–2018 period was referred to as Category 2, and the 2018–2023 period was called Category 3. Furthermore, to determine the changes between categories ‘1’, ‘2’ and ‘3’, category 1 was coded as ‘1’, Category 2 as ‘2’ and Category 3 was coded as ‘0’. A similar procedure was conducted to determine the changes among categories ‘1’ and 3’, categories ‘1’ and ‘2’ and categories ‘2 and 3’. In our analysis, survey mean command in STATA was used to compare each period and potential covariates (detailed results provided in Tables S1–S3). To determine the comparisons among the periods and report the significant differences, the linear combinations of parameters (lincom) command in STATA was used to determine the significance of differences at *p* < 0.05 for each of the potential covariates.

We performed bivariate analyses to assess the independent association between the outcome and exploratory variables. Furthermore, multiple logistic regression models were used to examine the factors associated with the three indicators of undernutrition among children under two years old in Tunisia. In the univariate analysis, all exploratory variables with a *p*-value < 0.20 were retained and used to build multiple logistic regression models. We used a manual elimination process for multiple logistic regression to eliminate non-significant variables (*p* > 0.05). Only those variables with *p* < 0.05 were considered factors associated with undernutrition among children under two years. The odds ratio (OR) and 95% confidence intervals were obtained from the adjusted logistic regression models and were used to determine factors associated with undernutrition among children under two years in Tunisia.

## 3. Results

### 3.1. Characteristics of the Participants

The current study had 1271, 1337 and 657 participants in the periods 2011–2012, 2017–2018 and 2022–2023, respectively, making a total of 3265 children aged 0–23 months. Across all three timeframes, urban dwellers outnumbered their rural counterparts. In all three time periods, the proportion of boys was approximately equal to the proportion of girls. The percentage of older mothers (35–49 years) was lower than that of younger mothers (15–34 years) in all three periods. Similarly, older fathers (45+ years) constituted the minority, compared with their younger counterparts, in all three timeframes. Across the three timeframes, married mothers completely outnumbered unmarried ones (90.7% versus 9.3% in 2011–2012, 92.7% versus 7.3% in 2017–2018 and 95.5% versus 4.5% in 2022–2023). Most parents (mothers and fathers) had received a school education, with only a small portion receiving none across the three time periods. Almost all households had access to clean cooking fuel in all three timeframes. Almost all households had access to the television across the three timeframes. Only a small proportion of children had contracted diarrhoea in all three time periods (12.2% versus 87.8% in 2011–2012, 12.2% versus 87.8% in 2017–2018 and 8.0% versus 92.0% in 2022–2023). Children were more likely to be delivered by skilled delivery assistants than by unskilled attendants during all three timeframes. More than two-thirds of mothers attended no postnatal clinics in all three timeframes. Across all three timeframes, less than 50% of mothers breastfed their babies within the first hour of delivery. More than 50% of babies were breastfed for more than 12 months in all three timeframes (see Table 1).

### 3.2. Prevalence and 95% CI of Undernutrition, 2011-12 to 2022-23

**Figure 1:**
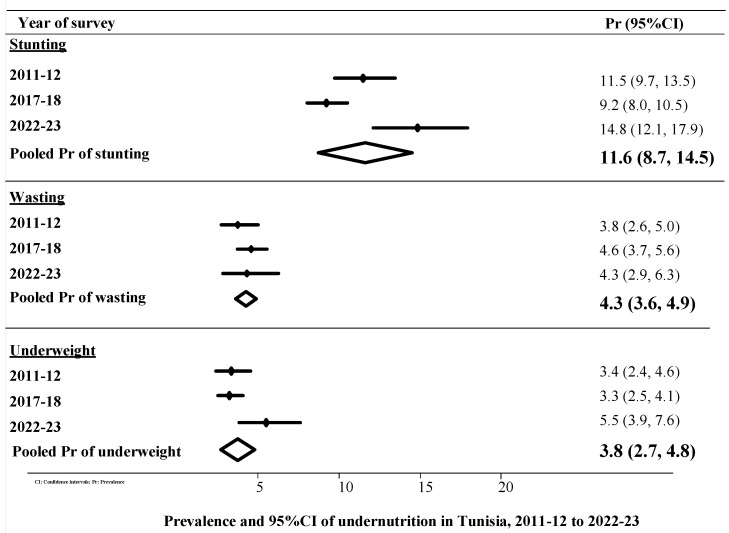
Prevalence of stunting, wasting and underweight by year of survey among children 0–23 months in Tunisia.

The overall prevalence of undernutrition (stunting, wasting, and underweight), as well as the 95% confidence intervals (CI) and the prevalence observed in each year, was examined (see Figure 1). The data reveal that the pooled prevalence of stunting over the past 10 years was 11.6%. Between 2011–2012 and 2022–2023, there was a noticeable rise in the prevalence of stunting, with an increase of approximately 3.3% over the course of the past decade. The observed increase did not reach statistical significance due to the overlapping 95% confidence intervals. The pooled prevalence of wasting was 4.3%, and over the course of the past ten years, there has been a 0.5% increase in the prevalence of wasting, with rates rising from 3.8% in 2011–2012 to 4.3% in 2022–2023. The overlap of the 95% confidence intervals indicates that the observed increase was not statistically significant. From 2012 to 2023, the overall prevalence of being underweight was 3.8%. Over the past decade, there has been a 2.1% increase in the prevalence of underweight, with the percentage rising from 3.4% in 2011–2012 to 5.5% in 2022–2023. Even though there was an increase, it was not considered statistically significant due to the overlapping 95% confidence intervals.

### 3.3. Trends in the Prevalence of Undernutrition from 2011–2012 to 2022–2023

The trends in the prevalence of stunting, wasting, and underweight from 2011–2012 to 2018 according to the investigated potential covariate factors are presented in Table 2. More details, including 95% confidence intervals, comparing the periods 2017–2018 and 2022–2023, can be found in Tables S1–S3.

In comparison to 2011–2012, the prevalence of stunting among urban children and middle wealth index households have increased by 7.0% and 8.1%, respectively, when compared to children born in 2022–2023. During the same timeframe, there were notable increases in stunted children across multiple factors. This includes children aged 0 to 5 months (11.8%), father with primary education (6.2%), children with mothers with no prior births (8.3%), children born to literate mothers (6.7%), no access to newspapers (6.0%), delivery at government health facilities (7.4%) and breastfed newborns within one hour after delivery (7.7%).

However, in comparison to the same timeframe, there was a significant decrease in stunting among Tunisian children under 2 years old among children with older mothers (aged 35–49 years) (−7.0%), and children with two or more siblings (−8.2%).

Although the prevalence of wasting remained relatively consistent across different factors, significant increases in underweight children were documented across multiple parameters from 2011–2012 to 2022–2023. This encompasses infants within the age range of 0 to 5 months (8.9%), fathers aged between 35 and 44 years (3.2%), fathers with only primary education (3.2%), mothers with no prior childbirths (4.1%), households lacking improved toilet facilities (4.3%), households without radio access (3.0%), households without newspaper access (4.4%), children born to literate mothers (5.5%) and duration of breastfeeding up to 12 months (7.4%).

### 3.4. Factors Associated with Undernutrition

The data in Table 3 indicate that in the univariate analysis, rural children were significantly more prone to stunting compared with their urban counterparts (unadjusted odds ratio (UOR) = 1.35; 95% confidence interval (CI): (1.06, 1.74)).

Compared with children from the richest households, the odds of being stunted were significantly lower among children from middle-class households (adjusted odds ratio (OR) = 0.52; 95% CI: (0.33, 0.82)). The likelihood of a child being stunted was significantly lower among those who were aged between 6 and 11 months, compared with infants who were aged 0–5 months (OR = 0.26; 95% CI: (0.18, 0.39)). In our univariate analysis, children were significantly more likely to be stunted when they had between four and eight members in their household, compared with children whose household consisted of between one and three members (UOR = 1.38; 95% CI: (1.04, 1.83)). The odds of being stunted were significantly higher among children whose mothers had no education compared with mothers who had secondary education or higher (OR = 2.06; 95% CI: (1.4, 2.92)). Children who had a mother with a BMI of more than 25 kgm^−2^ were significantly more prone to being stunted, compared with those whose mothers had a BMI of 18.5 kgm^−2^ or less (OR = 10.67; 95% CI: (5.97, 19.08)). The odds of being stunted were significantly more likely among children whose mothers put them to the breast within one hour after delivery compared with those who were put to the breast after one hour ((UOR) = 1.64; 95% CI: (1.28, 2.11)).

The study results demonstrated that the odds of wasting were significantly lower among females than their male counterparts (OR = 0.60; 95% CI: (0.37, 0.97)). Children who were aged 12–17 months had a significantly lower likelihood of wasting, compared with those aged 0–5 months (OR = 0.15; 95% CI: (0.07, 0.33)) (Table 2). The odds of wasting were significantly higher among children who had a mother that was older than 18 years at the time of marriage, compared with mothers 18 years old or younger at the time of marriage (OR = 10.12; 95% CI: (1.39, 73.51)). In the univariate analysis, the likelihood of wasting was significantly lower among children whose mother attended a post-natal care (PNC) clinic 2 days after delivery, compared with mothers who attended PNC 0–2 days after delivery (OR = 0.35; 95% CI: (0.17, 0.74)).

Females were significantly less likely to be underweight than their male counterparts (OR = 0.45; 95% CI: (0.27, 0.74)). The odds of being underweight were significantly less among children aged 12–17 months, compared with their counterparts who were aged 0–5 months (OR = 0.08; 95% CI: (0.03, 0.22)) (Table 2). The odds of underweight were significantly higher among children who were perceived to be small at birth, compared with infants who were of average size (OR = 1.90; 95% CI: (1.04, 3.47)). Children who had a mother with a BMI of between 19 and 25 kgm^−2^ were significantly less likely to be underweight, compared with mothers who had a BMI of 18.5 kgm^−2^ or less (OR = 0.23; 95% CI: (0.10, 0.54)). In the univariate analysis, the likelihood of underweight was significantly less among children who were breastfed for more than 12 months, compared with those who were breastfed for up to 12 months (OR = 0.42; 95% CI: (0.27, 0.65)).

## 4. Discussion

The current study utilised data from the Tunisian Multiple Indicator Cluster Surveys (MICS) from 2012 to 2023 to investigate the prevalence, trends, and associated factors contributing to undernutrition among Tunisian children aged 0–23 months. The study found an increase in the prevalence of the three indices of undernutrition—stunting, wasting, and underweight—among children under two years old in Tunisia over the last ten years. Specifically, stunting increased from 11.5% to 14.8%, wasting from 3.8% to 4.3%, and underweight from 3.4% to 5.5%. The multivariate analysis revealed that stunting, wasting, and being underweight were all associated with the year of the survey and the child’s age. The odds of stunting increased with high maternal BMI, decreased with families with middle wealth index, and the odds of being underweight increased with a mother with low BMI and a child with low birth size. Stunting trends increased among children aged 0–5 months, those with low-educated mothers, mothers aged 35–49 years, households with a middle wealth index, mothers with no previous births, mothers with high BMI, and those with late initiation of breastfeeding. While no significant trends were observed for factors associated with wasting, underweight trends were higher among children aged 0–5 months, those with fathers aged 33–45 years, mothers with no previous births, and those with a breastfeeding duration of up to 12 months.

In comparison to the UNICEF et al. 2023 levels and trends of child malnutrition report, our previous stated results show stunting among children in Tunisia was higher than the 2022 values for Algeria (8.6%) and Morocco (12.8%); but lower than those of the North Africa region (21.7%), Libya (52.2%) and Egypt (20.4%) [23]. The prevalence of wasting in Tunisia in 2023 was lower than the global value of 6.8% for the period 2015–2022, and that of the 2015–2022 value for North Africa (6.3%) [23], but higher than those of Algeria (2.7%) and Morocco (2.3%) for the same period. The prevalence of underweight in 2023 was higher than the 2019 values for Algeria (2.7%) and Morocco (2.8%), and lower than the 2014 values for Libya (11.7%) and Egypt (7.0%). The North African region struggles with undernutrition due to factors like disadvantage, inequality, and food insecurity, which are exacerbated by conflict, displacement, and climate change [13,14]. A recent UNICEF study found that at least 29 million children in the Middle East and North Africa live in poverty, with one in four children affected [24]. Countries like Algeria, Tunisia, and Libya experience significant disparities in poverty and unemployment rates based on age, gender, and location [25]. Economic inequality is a major obstacle to achieving the global goal of eradicating poverty by 2030 [26]. Africa ranks third in terms of significant disparities, after Latin America and the Middle East, due to economic, governmental, and social structures that limit access to healthcare, food, and other essential factors that impact children’s well-being [27].

The univariate and multivariate analyses highlight the strong connection between stunting and underweight in older children, demonstrating a continuation across the childhood development trajectory with an increase as children age. This is supported by prior research from low- and middle-income countries which has demonstrated an increase in stunting as children grow [3,28,29]. There is a critical window of growth when children are transitioning from exclusive breastfeeding to family foods, and their nutritional needs increase. Insufficient dietary intake, poor feeding practices, and limited access to nutrient-rich foods all contribute to an underweight nutritional status among children [10,30]. Although Tunisia experiences relatively low levels of hunger, economic hardships, especially reduced purchasing power, make it difficult for many families to afford nutritious food. The pandemic has further worsened the economic strain by severely impacting livelihoods. Combined with Tunisia’s vulnerability to climate change, which disrupts food availability and agriculture, these factors have significantly heightened the risks of food insecurity and persistent undernutrition [31]. In addition, the argument put forward by such studies is that after weaning a child from breastfeeding, normally at the second birthday the child may be considered old enough to be left under the care of their siblings or other caregivers, while their mother would be back to work, whether informal or formal, depending on her occupation [32]. Furthermore, from the available evidence, it is recognised that as children increase in age, there is an increased physiological requirement for optimal nutrient intake to support growth and development [33]. The data also suggest an improvement in diet, both in quantity and quality, which may not be possible depending on the financial status of the household [31]. Further studies comparing working and non-working mothers may provide better explanations for this association. In addition, a lack of access to sufficient nutrient-rich complementary foods to provide the child in the complementary feeding period, in addition to breast milk, can increase the risk of children acquiring early childhood infections [34,35,36]. This period is critical because children’s immune systems are still developing, and without proper nutrition, they become more susceptible to infections. Similar findings were reported in other studies, such as those conducted in Lalibela, Northern Ethiopia [37], and Indonesia [38], which also found a higher likelihood of stunting among children aged > 11 months.

On the other hand, the analysis discovered that children between the ages of 0 and 5 months were more prone to experiencing stunted growth when compared to their older counterparts. The discovery could be attributed to younger children being more prone to infection and illness, along with other factors that can impact the health and nutrition of women of reproductive age or pregnant women. These factors include birth length, maternal weight, birth order, maternal education levels, and wealth indicators [28]. According to the Tunisian Global Nutrition Report, the target of reducing anaemia among women aged 15 to 49 years has not been achieved, as 32.1% of them are still affected. Additionally, there has been no progress in reaching the low-birth-weight goal, as 7.5% of infants are still born with a low weight [39]. The UNICEF Data report highlighted the importance of delivering nutrition interventions during pregnancy and the first two years (first 1000 days) of a child’s life in Tunisia, in order to improve children’s chances of survival, development, and prevent stunting [40]. Scaling up nutrition intervention strategies, such as maternal nutrition and prenatal care education, promoting breastfeeding in community and facility settings, conditional cash transfers and related safety nets and innovative delivery strategies have the ability to prevent the occurrences of undernutrition in early ages [41].

The current study revealed that increased odds of stunting were significantly associated with middle household wealth index, compared to the richest households, which corroborates a finding from a past study that showed that household wealth was a significant predictor of health status, with relatively poorer households (middle index versus richest index) being reported to have poor nutritional, economic, environmental and health status [42]. It is therefore not surprising that relatively lower household index was a significant risk factor for stunting, as has been reported by other past studies [43,44,45]. The association between low household income and child stunting may be attributed to the fact that children from low-income households may have disadvantages of the likelihood to face food shortages, for instance, resulting in food insecurity, and inadequate feeding practices, which, in the end, may result in malnutrition and consequently lead to stunting [9,46]. In order to address child undernutrition in low-income households, the World Health Organization recommends that infants begin complementary feeding alongside breast milk at six months of age [47]. Infants should be complementarily fed two to three times per day between 6 and 8 months, and three to four times per day from 9 to 11 months through to 24 months, to meet their growing nutritional needs [47]. This gradual increase in feeding helps reduce the risk of malnutrition and stunting, particularly in poorer households. Additionally, low-income families often face challenges related to poor maternal nutrition, which has been identified as a critical risk factor for child health outcomes [9,46,48]. Many women in low-income households consume diets deficient in essential minerals such as iodine, iron, folate, calcium, and zinc [49]. During pregnancy, these deficiencies can lead to adverse child outcomes, such as low birth weight and developmental delays, as observed by UNICEF, which reports that over 20 million newborns are affected by low birth weight each year [49]. Poor maternal nutrition during breastfeeding further diminishes a mother’s ability to meet her nutritional needs, often constrained by limited access to and affordability of nutritious food. This restriction significantly affects women’s capacity to make healthy nutritional choices, contributing to the cycle of malnutrition and poor health outcomes for both mother and child [49].

The study analysis showed that an increased risk of stunting was associated with maternal obesity, meaning that children whose mothers were overweight were more prone to being stunted. It has been established in past research that, worldwide, the coexistence of maternal obesity and child stunting (Mother-Child-Double-Burden) is connected to undernutrition during early life and exacerbated by the nutrition transition [50,51,52]. A lack of essential nutrients in calorie-dense diets can result in both obesity and stunting. Several low- and middle-income countries rely heavily on ultra-processed, low-nutritional meals heavy in fats and sugars, causing adults to gain weight, while children, particularly during critical growth phases, suffer from nutrient deficits that result in stunted growth [53]. Furthermore, women who have excess weight have lower rates of breastfeeding than those who are not overweight. A two-cohort longitudinal study conducted at Oregon Health & Science University, USA, found that women with a pre-pregnancy BMI ≥30 kg/m^2^ have a lower intention to exclusively breastfeed than those of normal weight and overweight (78.8% vs. 95.5% and 96.2%, respectively) [54]. Similar findings were found in both a systematic investigation focusing on the North African region [13] and a cross-sectional study conducted in Nepal [55]. The study discovered a significant association between child stunting and having an overweight mother. However, findings demonstrated that infants born to mothers with low BMI are more likely to be underweight. A research study conducted in Tanzania [56] examined children aged 0–23 months and discovered similar outcomes in terms of the probability of a child being underweight when the mother’s BMI was below 18.5 kgm^−2^ [56]. Furthermore, the previous studies conducted in Rwanda [57] and Ethiopia [58] have confirmed the significant correlation identified between underweight and a mother’s normal or low BMI. The finding could be related to the fact that the physiological impacts of a mother’s malnutrition during lactation may have an impact on milk secretion and the child’s weight gain. To address the double burden of malnutrition in the family, interventions should include increased nutrition, education, and healthcare, to enhance both maternal and child health [59,60].

Low birth weight is a critical indicator of a baby’s immediate and future health. LBW babies face higher risks of infant mortality and illness, poor cognitive development, and chronic diseases like diabetes and cardiovascular disease later in life [61]. Additionally, they are at higher risk of developmental difficulties and poor motor skills [62], with increased mortality rates for those with very low birth weight [63]. The study multivariate analysis indicated that children born with a small size are more likely to be underweight compared to those with normal or larger birth sizes. This is consistent with findings from a recent systematic review and meta-analysis focused on North Africa [13] and a study based on the Malawi Demographic and Health Survey [64]. The link between low birth weight (LBW) and childhood undernutrition is often mediated by increased infection rates in LBW children. Malnutrition impacts the immune system, making children more susceptible to infections, which further exacerbates malnutrition [65,66].

The multivariate analysis found that only child age, wealth index, birth size, and mother’s body mass index (BMI) were significantly linked to undernutrition in Tunisian children younger than two years. However, looking at the study trends showed that some socioeconomic factors have become more significant over the past ten years. The observed increase in undernutrition among Tunisian children appears to be linked to these factors, highlighting the necessity for attention and intervention. The following provides a detailed discussion of some of these factors:

The percentage of stunted urban children increased dramatically between 2011–2012 and 2022–2023, showing an improvement in rural patterns in undernutrition over that time period. Previous research conducted in Maharashtra, India [67] and sub-Saharan nations [68] revealed similar findings. The trend in urban-rural differentials is primarily due to stagnant trends in urban undernutrition combined with improvements in rural housing. A variety of variables may have contributed to trend improvements in rural areas. One such effect could be Tunisia’s large growth in food production over that time span [69]. Since the majority of the food consumed in Tunisia is produced in the rural areas, it is conceivable that there was increased availability of food supplies in rural households during that period; and the availability of food in the household would imply that the children would be adequately fed. Another possible reason for the urban-rural disparities is that poverty is predominantly seen as a rural issue in Tunisia [69]. Consequently, the majority of interventions aimed at alleviating poverty are targeted towards rural populations [70]. These interventions include programs like cash transfers, nutritional supplementation, initiatives to improve food security and social protection, as well as programs to enhance health services coverage [28,71]. These interventions have the potential to enhance child health in rural and disadvantaged areas.

The study results showed that children whose fathers had only primary education experienced a notable rise in the rates of stunting and underweight. Although these factors may not be primary drivers of the growing undernutrition issue in Tunisia, they warrant attention due to their notable increase among undernourished children in the country in recent times, and therefore should be taken into consideration. Previous studies found that lower maternal and paternal education levels were associated with childhood undernutrition [13,72,73]. A study in Libya, which shares cultural and societal similarities with Tunisia, found a significant link between a father’s educational level and the prevalence of stunting in children, as revealed by The Libyan Maternal and Child Health Survey [74]. This finding has implications for understanding the factors contributing to stunting in Tunisia as well. In Arabic patrilineal cultures, fathers play a vital role in preventing child undernourishment, and their involvement from pregnancy to early childhood is crucial for healthy growth and development. As primary providers, fathers are essential in ensuring family nutrition and preventing undernutrition. Their participation in household management also contributes to improved child nutrition. While maternal education is often emphasized, in Arabic societies, fathers are primarily responsible for family well-being. The interconnectedness of parental education and marital decisions among educated women makes it challenging to distinguish the separate effects of maternal and paternal education on child health. However, educated families tend to have better living conditions, healthcare access, and hygiene practices, ultimately benefiting their children’s health [75,76].

The study analysis revealed an increased prevalence of stunting with children whose mothers put them to the breast within one hour after delivery, which was inconsistent with a finding in a past study which revealed that children who are not provided with an early initiation of breastfeeding are 1.3 times more likely to be stunted compared with those who are breastfed early [77]. In fact, early initiation of breastfeeding typically does not lead to an increase in stunting rates. It is possible that, in certain circumstances, factors such as inadequate maternal nutrition, substandard breastfeeding techniques, or increased illness frequencies could continue to affect stunting, despite the prompt onset of breastfeeding [1]. The early initiation of breastfeeding is usually associated with enhanced growth outcomes rather than an increased risk of stunting due to the provision of essential nutrients through immune-boosting colostrum [1,10].

Furthermore, there was a higher trend of underweight among children who had duration of breastfeeding up to 12 months. The potential rationale for breastfeeding for a maximum of 12 months may be linked to non-compliance with the WHO’s recommended duration of 2 years for breastfeeding; and this may be attributed to the mother’s occupational commitments and insufficient time to take care of her child [78]. Alternatively, it could be a result of insufficient complementary feeding and cessation of breastfeeding [34,36,78]. The aforementioned factors demonstrated a notable rise among children below 2 years of age in Tunisia, despite the absence of any correlation in the study’s multivariate analysis. Nevertheless, it is crucial for Tunisian government policymakers and public health researchers to consider these factors, as they have become more pronounced alongside the rise in stunting and underweight cases in 2023. The current study identified additional factors contributing to undernutrition among children under two in Tunisia, including maternal age (35–49 years), first-time mothers, paternal age (33–45 years), and breastfeeding duration of up to 12 months.

It is crucial to remember that Tunisia’s situation has worsened due to a combination of factors, including political instability, widespread poverty and inequality, natural disasters intensified by climate change, and the recent pandemic [6,7,8]. These challenges can exacerbate maternal and child health problems, placing an increased burden on vulnerable communities by restricting access to maternal and child healthcare services, food, and essential resources such as clean water and sanitation, worsening the prevalence of malnutrition and disease. In Tunisia, the economic crisis subsequent to the COVID-19 pandemic has impeded the progress achieved in poverty reduction over recent decades [79]. The national poverty line in Tunisia reveals a decline in official poverty rates from 20.5% in 2010 to 15.2% in 2015, followed by an increase to 16.6% in 2021 [79]. Moreover, the country experiences significant disparities in socioeconomic conditions, both within urban and rural areas, and across different regions [79]. Poverty and equality are key factors associated with the deterioration of mother and child health [42,50]. Tunisia’s political transition has been successful, but its economic development has been slow. The country faces challenges like political fragmentation and a lack of agreement on essential economic changes. The influence of civil wars in neighbouring countries, especially the ongoing conflict in Libya, has hindered the economic recovery and worsened social unrest and youth unemployment [80].

To effectively address undernutrition in Tunisian children, it is essential to concentrate on the following critical areas: (i) Maternal health and education: Enhancing maternal health, parental education, and access to prenatal care for the early detection and prevention of undernutrition. (ii) Promoting breastfeeding: Emphasis should be placed on promoting early initiation of breastfeeding and extended breastfeeding, alongside adequate complementary nutrition, especially for underprivileged families. (iii) The Tunisian government is responsible for improving infant nutrition by creating an appropriate environment, which includes implementing policies that enhance food security, ensuring access to healthcare services, providing nutrition education, and providing resources for families to maintain healthy diets [28,71]. (iv) Public Health Initiatives: Effective public health strategies are essential for combating undernutrition. Essential programs must include community-based initiatives focused on combating child undernutrition, promoting breastfeeding, and nutritional assistance initiatives, such as nutrition education programs. (v) Cross-Sectoral Collaboration: Addressing undernutrition requires collaboration across multiple sectors, including health, agriculture, education, environment, community groups, and the nutrition industry [28,71].

The current study has several strengths. First, it is nationally representative and population-based, with a large sample size which yielded high response rates for children and mothers/caregivers. Secondly, the study utilised the Tunisia 2012, 2018 and 2023 MICS dataset, which were the most recent nationally recognised data available in Tunisia, thus making the study relevant. Third, suitable statistical adjustments were applied to the three MICS datasets, and the most vulnerable subpopulation affected by stunting, wasting and underweight was identified. Nonetheless, the study has some limitations. First, due to the cross-sectional nature of the study design, the causal relationship among the observed risk factors and the dependent variables could not be established. Second, the effect of residual confounding as a result of unmeasured co-variates could not be ruled out, in spite of using a comprehensive set of variables in the analysis; this took into consideration direct measures of children’s diet, Vitamin A supplementation, and feeding patterns, as well as energy expenditure, through physical activity, in the identification of possible casual paths.

## 5. Conclusions

Over the past decade, Tunisia has seen a rise in key indicators of undernutrition—stunting, wasting, and underweight—among children under two. The growing prevalence of factors contributing to child undernutrition is a significant concern, demanding an immediate and coordinated response. Community-based interventions are essential for improving child health and nutrition, particularly through nutrition interventions during pregnancy and the first two years of life, which are crucial for children’s survival and development. Efforts should include financial support for nutritional supplementation, initiatives to improve food security and social protection, and expanded healthcare coverage. Parental education programs, especially for first-time mothers, are vital in promoting proper feeding practices. These programs should emphasize maternal nutrition during pregnancy, increase access to nutrient-dense foods for mothers and children, and provide breastfeeding counselling particularly for mothers with overweight in the postnatal period. To achieve sustained improvements in child health outcomes, policies addressing food security, healthcare access, and nutrition education must be prioritized by public health authorities, the government, and stakeholders.

## Figures and Tables

**Table 1:** Distribution of determinants of child nutritional health: Tunisia Multiple Indicator Cluster Surveys for the years 2011–2012, 2017–2018, and 2022–2023.

	Years 2011–2012 (n = 1271)	Years 2017–2018 (n = 1337)	Years 2022–2023 (n = 657)
Area of residence	Total n (%)	Total n (%)	Total n (%)
Urban	808 (63.56)	831 (62.15)	366 (55.77)
Rural	463 (36.44)	506 (37.85)	291 (44.23)
Wealth Index			
Poorest	344 (27.06)	278 (20.79)	116 (17.6)
Poorer	295 (23.2)	175 (13.12)	191 (29.13)
Middle	245 (19.26)	257 (19.24)	143 (21.73)
Fourth	160 (12.6)	391 (29.23)	88 (13.4)
Richest	227 (17.88)	235 (17.61)	119 (18.13)
Sex of baby			
Boy	678 (53.37)	726 (54.33)	352 (53.64)
Girl	592 (46.63)	610 (45.67)	305 (46.36)
Child age (months)			
0–5	337 (26.49)	316 (23.65)	150 (22.77)
6–11	330 (25.99)	321 (24.02)	147 (22.36)
12–17	302 (23.76)	338 (25.26)	166 (25.29)
18–23	302 (23.76)	362 (27.08)	194 (29.58)
Mother’s age (years)			
15–34	938 (74.05)	901 (67.88)	391 (60.99)
35–49	329 (25.95)	426 (32.12)	250 (39.01)
Father’s age (years)			
18–34	380 (33.11)	374 (30.4)	172 (28.04)
35–44	598 (52.1)	656 (53.3)	320 (52.28)
45+	170 (14.79)	201 (16.3)	121 (19.68)
Mother’s age at marriage (years)			
≤18 years	44 (3.79)	32 (2.55)	27 (4.33)
>18 years	1118 (96.21)	1207 (97.45)	590 (95.67)
Marital status			
Married	1148 (90.69)	1231 (92.73)	613 (95.45)
Not married	118 (9.31)	96 (7.27)	29 (4.55)
Maternal education			
Secondary and above	771 (60.91)	973 (73.29)	188 (30.51)
Primary	354 (27.93)	275 (20.73)	339 (55.18)
No education	141 (11.16)	79 (5.98)	88 (14.31)
Father education			
Secondary and above	751 (59.12)	950 (71.1)	193 (31.09)
Primary	370 (29.9)	288 (21.58)	334 (53.82)
No education	150 (11.8)	98 (7.33)	94 (15.09)
Maternal BMI (kgm^−2^)			
≤18.5	750 (64.12)	878 (66.71)	390 (65.1)
19–25	380 (32.52)	419 (31.84)	182 (30.34)
25+	39 (3.37)	19 (1.45)	27 (4.56)
Household members			
1–3	311 (24.45)	650 (48.65)	116 (17.67)
4–8	855 (67.31)	677 (50.67)	506 (76.97)
>8	105 (8.24)	9 (0.68)	35 (5.36)
Birth order			
None previous	474 (37.28)	770 (57.6)	352 (53.51)
1	601 (47.3)	354 (26.52)	205 (31.19)
2 or more	196 (15.43)	212 (15.88)	101 (15.31)
Cooking fuel			
Clean	1270 (99.91)	1333 (99.84)	656 (99.83)
Unclean	1 (0.09)	2 (0.16)	1 (0.17)
Source of drinking water			
Protected	497 (62.74)	725 (54.23)	354 (53.9)
Unprotected	474 (37.26)	612 (45.77)	303 (46.1)
Toilet facility			
Improved	784 (61.72)	555 (41.53)	298 (45.32)
Unimproved	486 (38.28)	781 (58.47)	359 (54.68)
Listening to the radio			
Not at all	569 (44.9)	722 (54.38)	319 (49.78)
Yes	698 (55.1)	606 (45.62)	321 (50.22)
Watching TV			
Not at all	22 (1.72)	3 (2.33)	31 (4.91)
Yes	1245 (98.28)	1296 (97.67)	609 (95.09)
Reading newspaper			
Not at all	464 (42.9)	105 (79.2)	523 (81.57)
Yes	617 (57.1)	276 (20.8)	118 (18.43)
Size of baby			
Average	824 (72.69)	852 (69.85)	418 (68.63)
Small	161 (14.23)	154 (12.65)	94 (15.4)
Large	148 (13.08)	213 (17.5)	97 (15.97)
Diarrhoea last two weeks			
No	1115 (87.85)	1173 (87.8)	600 (92.03)
Yes	154 (12.15)	163 (12.2)	52 (7.97)
Fever			
No	265 (20.83)	1025 (76.82)	542 (82.75)
Yes	1006 (79.17)	309 (23.18)	113 (17.25)
Cough			
No	682 (53.74)	934 (69.91)	492 (75.18)
Yes	587 (46.26)	402 (30.09)	163 (24.82)
Any infection			
No	137 (10.8)	762 (57.03)	444 (67.63)
Yes	1134 (89.2)	574 (42.97)	213 (32.37)
Place of delivery			
Government	345 (27.14)	1217 (99.73)	604 (98.85)
Non-government	926 (72.86)	3 (0.27)	7 (1.15)
Antenatal clinic visits			
8+	391 (30.78)	304 (22.72)	165 (25.26)
4–7	571 (44.92)	723 (54.06)	315 (48.15)
1–3	142 (11.18)	139 (10.43)	79 (12.06)
None	167 (13.12)	171 (12.79)	95 (14.53)
Delivery assistance			
Skilled	831 (65.4)	1215 (90.92)	601 (91.48)
Unskilled	440 (34.6)	121 (9.08)	56 (8.52)
Mode of delivery			
Non-caesarean	16 (1.41)	683 (56.12)	325 (54.05)
Caesarean	1113 (98.59)	534 (43.88)	276 (45.95)
Postnatal checkup (days)			
0–2	38 (3.02)	180 (13.45)	125 (18.98)
After 2	360 (28.32)	206 (15.38)	83 (12.65)
No	872 (68.66)	951 (71.17)	449 (68.36)
Early initiation of breastfeeding			
After 1 h	753 (59.26)	858 (64.23)	411 (62.49)
Within 1 h	518 (40.74)	478 (35.77)	246 (37.51)
Ever breastfeed			
Yes	555 (43.66)	1135 (84.9)	561 (85.39)
No	716 (56.34)	202 (15.1)	96 (14.61)
Duration of breastfeeding (months)			
Up to 12	317 (24.95)	564 (42.21)	280 (42.56)
>12	954 (75.05)	772 (57.79)	377 (57.44)
Literacy			
Yes	309 (62.6)	223 (17.85)	346 (56.47)
No	184 (37.4)	1026 (82.15)	266 (43.53)

**Table 2:** Trends in prevalence of stunting, wasting and underweight among children aged 0–23 months in Tunisia, 2011–2012 to 2022–2023.

Characteristics	Stunting			Wasting			Underweight		
	2011–2012	2022–2023	2011–2012–2022–2023	2011–2012	2022–2023	2011–2012–2022–2023	2011–2012	2022–2023	2011–2012–2022–2023
	%	%	%	%	%	%	%	%	%
Place of residence									
Urban	9.3	16.4	7.0 **	2.9	4.8	1.8	2.9	6.2	3.3
Rural	15.1	12.8	−2.4	5.2	3.6	−1.7	4.2	4.7	0.5
Wealth Index									
Poorest	8.9	21.3	12.3	4.0	9.2	5.2	3.8	12.1	8.3
Poorer	14.3	15.5	1.3	4.6	3.4	−1.2	3.2	4.3	1.1
Middle	4.6	12.7	8.1 **	2.3	4.9	2.6	2.2	7.2	5.1
Fourth	14.1	9.2	−4.9	5.2	1.7	−3.6	2.6	1.6	−0.9
Richest	17.1	14.4	−2.7	3.2	2.3	−0.8	4.8	2.3	−2.5
Sex of baby									
Boy	12.9	16.5	3.7	4.8	6.3	1.5	4.7	7.5	2.8
Girl	9.9	12.8	2.9	2.7	2.0	−0.7	1.8	3.2	1.4
Child age (months)									
0–5	16.0	27.8	11.8 **	6.6	8.2	1.6	6.9	15.7	8.9 **
6–11	5.1	7.8	2.7	4.4	1.5	−2.9	2.6	4.6	2.0
12–17	15.5	13.6	−1.9	1.4	1.5	0.2	0.7	2.3	1.7
18–23	9.3	10.9	1.6	2.3	5.5	3.21	3.0	1.2	−1.8
Mother’s age (years)									
15–34 years	11.1	17.2	−0.4	3.9	4.2	0.3	3.7	6.6	2.9
35–49 years	12.5	11.5	−7.0 **	3.4	4.2	0.8	2.5	3.7	1.2
Father’s age (years)									
18–34	10.5	10.5	4.9	4	5.4	1.4	6.4	5.7	−0.7
35–44	11.5	11.5	2.8	3.4	3.7	0.3	2.0	5.2	3.2 **
45+	12.0	12.0	5.3	3.9	4.3	0.4	3.1	6.5	3.4
Mother’s age at marriage (years)									
≤18	12.6	12.5	−0.1	0.9	0.0	−0.9	-	-	9.2
>18	11.4	15.4	4.0	4	4.4	0.4	3.7	5.4	1.7
Marital status									
Married	11.3	15.2	3.9	3.7	4.3	0.6	3.6	5.6	2.0
Not married	13.8	10.8	−3.0	4.7	2.7	−2.1	1.0	2.5	1.5
Maternal education									
Secondary and above	10.1	13.1	3.05	3.8	0.6	2.2	3.9	5.8	1.9
Primary	11.0	16.3	5.2	3.3	3.4	0.2	1.6	4.0	2.4
No education	20.0	16.7	−3.3	5.2	2.1	−3.1	4.9	11.4	6.5
Father education									
Secondary and above	10.1	13.1	3.05	4.1	6.3	2.3	3.9	5.4	1.5
Primary	10.0	16.2	6.2 **	2.8	3.7	0.9	1.5	4.7	3.2 **
No education	21.6	15.4	−6.3	4.9	1.2	−3.6	5.4	10.0	4.6
Maternal BMI (kgm^−2^)									
≤18.5	6.3	8.5	2.2	5.9	6.5	0.6	4.6	6.6	2.0
19–25	19.7	23.6	3.9	-	-	-	1.0	3.9	2.9
25+	32.0	50.2	18.2	-	-	-	2.4	-	−2.4
Household members									
1–3	9.8	11.3	1.51	6.3	10.2	3.9	4.5	3.5	−1.0
4–8	12.3	15.9	3.62	2.7	3.0	0.3	3.3	6.0	2.7
>8	10.2	9.7	−0.49	4.9	3.0	−1.9	1.0	5.5	4.5
Birth order									
None previous	10.4	18.6	8.3 **	5.4	5.3	−0.1	3.3	7.4	4.1 **
1	11.3	12.0	0.8	3.1	2.3	−0.7	3.3	2.0	−1.3
2 or more	14.8	6.6	−8.2 **	2.1	4.1	1.9	3.9	5.6	1.7
Source of drinking water									
Protected	11.6	13.9	2.3	3.7	4.2	2.0	4.0	5.3	1.3
Unprotected	11.3	15.8	4.5	4.0	4.3	−0.01	2.4	5.7	3.3
Toilet facility									
Improved	11.6	12.7	1.1	3.7	4.7	2.0	4.0	4.0	−0.1
Unimproved	11.3	16.5	5.2	3.9	3.9	−0.01	2.4	6.8	4.3 **
Listening to the radio									
Not at all	10.6	15.2	4.6	3.1	4.1	1.0	2.4	5.4	3.0 **
Yes	12.2	14.9	2.7	4.4	4.4	0.2	4.2	5.6	1.3
Watching TV									
Not at all	20.1	25.8	5.7	-	3.8	−1.0	2.9	8.4	5.6
Yes	11.3	14.4	3.1	3.9	4.2	0.1	3.4	5.3	1.9
Read newspaper									
Not all	9.0	15.0	6.0 **	2.0	4.5	2.5	1.7	6.1	4.4 **
Yes	10.9	15.3	4.4	4.9	2.9	−2.0	4.2	2.6	−1.7
Size of baby									
Average	11.6	16.2	4.6	3.7	5.1	1.4	3.6	5.1	1.6
Small	13.7	14.3	0.6	6.3	3.6	−2.7	7.0	5.6	−1.5
Large	8.7	12.5	3.8	1.4	1.3	−0.1	0.5	7.8	7.3 **
Diarrhoea last two weeks									
No	11.6	14.6	3.0	3.8	4.5	0.7	3.5	5.6	2.2
Yes	11.0	17.4	6.4	3.7	1.9	−1.9	2.9	4.8	1.9
Fever									
No	15.5	15.8	0.28	4.5	4.4	−0.1	3.3	6.1	2.8
Yes	10.4	10.3	−0.13	3.6	3.5	−0.1	3.4	2.5	−0.9
Cough									
No	10.4	16.1	5.7 **	4.8	4.3	−0.5	4.5	5.9	1.4
Yes	12.7	11	−1.8	2.6	4.0	1.4	2.0	4.4	2.4
Any infection									
No	13.4	15.3	1.9	6.6	4.4	−2.3	5.3	6.0	0.7
Yes	11.2	13.6	2.4	3.4	4.0	0.5	3.1	4.4	1.3
Place of delivery									
Government	7.9	15.3	7.4 **	3.9	3.2	−0.6	3.0	4.4	1.3
Non-government	7.3	15.3	8.0	3.7	7.5	3.8	4.1	9.5	5.4
Antenatal clinic visits									
8+	14.3	19.9	5.6	3.2	7.1	3.9	4.0	3.9	−0.2
4–7	9.6	13.7	4.12	5.2	3.2	−2.0	4.0	6.8	2.8
1–3	11.3	14.3	2.1	0.3	2.3	2.0	1.4	3.9	2.5
None	11.2	10.1	−1.1	3.3	4.7	1.4	1.1	5.3	4.2
Delivery assistance									
Skilled	11.0	15.3	4.3 **	3.8	4.4	0.6	3.8	5.7	1.9
Unskilled	12.4	8.5	−3.9	3.8	2.9	−0.9	2.6	2.9	0.3
Mode of delivery									
Non-caesarean	11.2	17.2	6.0 **	3.6	4.0	0.4	3.7	5.7	2.0
Caesarean	11.5	13.6	2.0	3.9	4.8	0.9	3.4	5.8	2.4
Postnatal checkup									
0–2 days	14.0	16.5	2.6	13	7.7	−5.3	9.5	0.9	−8.6
After 2 days	13.2	21.6	8.4	2.2	4.3	2.1	3.4	8.1	4.8
No	10.6	13.1	2.5	4.1	3.3	−0.8	3.1	6.3	3.1
Early initiation of breastfeeding									
After 1 h	9.4	10.3	0.97	4.0	4.3	0.3	2.9	5.8	2.9
Within 1 h	14.5	22.2	7.7 **	3.4	4.1	0.7	4.0	5.0	1.0
Ever breastfeed									
Yes	13.9	16	−2.3	3.1	3.5	0.4	3.8	6.0	2.2
No	9.5	7.2	2.1	4.4	8.7	4.3	3.1	2.7	−0.4
Duration of breastfeeding (months)									
Up to 12	13.5	17.9	5.4	4.1	3.6	−0.5	4.7	9.7	7.4 **
>12	10.8	12.5	2.7	3.7	4.7	1.0	2.9	2.3	1.7
Literacy									
Yes	10.2	16.9	6.7 **	3.1	3.8	0.7	1.2	6.6	5.5 **
No	19.3	13.6	−5.7	4.7	4.4	−0.30	4.9	4.4	−0.5

− Omitted values; ** *p* < 0.01.

**Table 3:** Unadjusted and adjusted odds ratios for determinants of indicators of stunting, wasting and underweight among children aged 0–23 months in Tunisia.

Characteristics	Stunting				Wasting				Underweight			
	Unadjusted	*p*	Adjusted	*p*	Unadjusted	*p*	Adjusted	*p*	Unadjusted	*p*	Adjusted	*p*
Years of survey												
2012	1		1				1		1		1	
2018	0.72 [0.54, 0.96]	0.023	0.73 [0.52, 1.01]	0.054	0.78 [0.45, 1.25]	0.279	0.79 [0.47, 1.34]	0.388	0.74 [0.44, 1.24]	0.253	0.70 [0.41, 1.20]	0.191
2023	1.34 [0.96, 1.87]	0.088	1.22 [0.85, 1.74]	0.284	1.12 [0.63, 1.10]	0.689	1.16 [0.64, 2.12]	0.620	1.66 [0.95, 2.91]	0.074	1.70 [0.94, 3.10]	0.082
Area of residence												
Urban	1				1				1			
Rural	1.35 [1.06, 1.74]	0.017			1.08 [0.69, 1.67]	0.745			1.09 [0.70, 1.69]	0.717		
Wealth Index												
Poorest	1		1		1				1			
Poorer	1.36 [0.92, 2.02]	0.128	1.27 [0.84, 1.91]	0.257	0.98 [0.50, 1.93]	0.958			0.78 [0.42, 1.44]	0.424		
Middle	0.56 [0.37, 0.86]	0.008	0.52 [0.33, 0.82]	0.004	0.89 [0.45, 1.74]	0.726			0.65 [0.32, 1.35]	0.248		
Fourth	0.79 [0.53, 1.18]	0.250	0.83 [0.54, 1.28]	0.395	0.73 [0.35, 1.50]	0.391			0.43 [0.21, 0.88]	0.022		
Richest	1.20 [0.82, 1.76]	0.349	1.29 [0.87, 1.92]	0.210	0.82 [0.41, 1.64]	0.580			0.66 [0.35, 1.24]	0.199		
Sex of baby												
Boy	1				1		1		1		1	
Girl	0.85 [0.66, 1.10]	0.212			0.61 [0.39, 0.96]	0.034	0.60 [0.37, 0.97]	0.036	0.47 [0.29, 0.76]	0.002	0.45 [0.27, 0.74]	0.002
Child age												
0 to 5	1		1		1		1		1		1	
6 to 11	0.35 [0.24, 0.52]	<0.001	0.26 [0.18, 0.39]	<0.001	0.52 [0.24, 1.76]	0.004	0.42 [0.23, 0.77]	0.006	0.45 [0.14, 0.44]	<0.001	0.20 [0.10, 0.38]	<0.001
12 to17	0.62 [0.45, 0.87]	0.006	0.44 [0.31, 0.63]	<0.001	0.14 [0.07, 0.31]	<0.001	0.15 [0.07, 0.33]	<0.001	0.08 [0.03, 0.23]	<0.001	0.08 [0.03, 0.22]	<0.001
18–23	0.58 [0.42, 0.81]	<0.001	0.47 [0.33, 0.66]	<0.001	0.32 [0.18, 0.58]	<0.001	0.32 [0.17, 0.59]	<0.001	0.22 [0.12, 0.40]	<0.001	0.22 [0.12, 0.42]	<0.001
Father’s age (years)												
18–34	1				1				1			
35–44	1.24 [0.91, 1.69]	0.174			0.71 [0.43,1.19]	0.196			0.54 [0.33, 0.88]	0.014		
45+	1.25 [0.85, 1.85]	0.259			0.78 [0.39, 1.58]	0.497			0.76 [0.39, 1.50]	0.434		
Mother’s age at marriage (years)												
≤ 18	1				1		1		1			
>18	1.40 [0.50, 2.14]	0.916			10.68 [1.46, 78.0]	0.020	10.12 [1.39,73.51]	0.022	1.57 [0.22, 11.48]	0.656		
Birth order												
None previous	1				1				1			
1	0.87 [0.66, 1.16]	0.347			0.76 [0.47, 1.23]	0.263			0.62 [0.36, 1.07]	0.085		
2 or more	1.08 [0.77, 1.52]	0.663			0.56 [0.26, 1.19]	0.132			1.19 [0.69, 2.09]	0.532		
Household members												
1–3	1				1				1			
4–8	1.38 [1.04, 1.83]	0.024			0.53 [0.34, 0.84]	0.006			1.27 [0.78, 2.07]	0.344		
>8	1.12 [0.62, 2.02]	0.704			0.95 [0.38, 2.35]	0.910			0.82 [0.32, 2.07]	0.672		
Size of baby												
Average	1				1				1		1	
Small	1.34 [0.95, 1.88]	0.092			1.48 [0.82, 2.68]	0.197			1.86 [1.04, 3.31]	0.035	1.90 [1.04, 3.47]	0.037
Large	0.58 [0.36, 0.94]	0.026			0.53 [0.25, 1.11]	0.093			0.73 [0.36, 1.47]	0.377	0.65 [0.31, 1.34]	0.241
Source of drinking water												
Protected	1				1				1			
Unprotected	0.95 [0.74, 1.23]	0.706			1.45 [0.93, 2.25]	0.098			1.07 [0.68, 1.67]	0.773		
Toilet facility												
Improved	1				1				1			
Unimproved	1.03 [0.80, 1.32]	0.812			1.26 [0.81, 1.96]	0.306			1.26 [0.81, 1.96]	0.306		
Listening to the radio												
Not at all					1				1			
Yes	0.07 [0.83, 1.37]	0.618			1.13 [0.73, 1.75]	0.598			1.34 [0.87, 2.08]	0.189		
Watching TV												
Not at all	1				1				1			
Yes	0.49 [0.29, 0.84]	0.009			2.59 [0.35, 18.86]	0.349			0.43 [0.17, 1.08]	0.072		
Read newspaper												
Not at all	1				1				1			
Yes	1.0 [0.75, 1.35]	0.969			1.22 [0.75, 1.99]	0.418			0.98 [0.59, 1.61]	0.925		
Maternal education												
Secondary and above	1				1				1			
Primary	1.37 [1.03, 1.82]	0.033			0.93 [0.56, 1.56]	0.780			0.84 [0.50, 1.40]	0.504		
No education	2.06 [1.4, 2.92]	<0.001			1.15 [0.59, 1.26]	0.678			1.96 [1.04, 3.69]	0.036		
Maternal BMI												
≤18.5	1				-				1		1	
19–25	3.02 [2.32, 3.93]	<0.001	3.51 [2.69, 4.58]	<0.001	-				0.28 [0.15, 0.52]	<0.001	0.23 [0.10, 0.54]	0.001
25+	9.27 [5.35, 16.04]	<0.001	10.67 [5.97,19.08]	<0.001	-				0.24 [0.03, 1.73]	0.156	0.20 [0.03, 1.61]	0.13
Place of delivery												
Government	1				1				1			
Non-government	1.07 [0.66, 1.73]	0.780			1.73 [0.83, 3.58]	0.143			2.23 [1.14, 4.37]	0.020		
Delivery assistance												
Skilled	1				1				1			
Unskilled	1.06 [0.78, 1.43]	0.708			0.90 [0.51, 1.60]	0.727			0.71 [0.40, 1.26]	0.241		
Antenatal clinic visits												
8+	1				1				1			
4–7	0.70 [0.52, 0.95]	0.020			0.73 [0.44, 1.22]	0.232			1.23 [0.72, 2.11]	0.456		
1–3	0.73 [0.47, 1.11]	0.143			0.57 [0.26, 1.25]	0.158			1.01 [0.48, 2.11]	0.987		
None	0.78 [0.53, 1.17]	0.231			0.62 [0.30, 1.30]	0.204			0.97 [0.47, 1.10]	0.925		
Postnatal checkup												
0–2 days	1				1				1			
After 2 days	0.91 [0.57, 1.45]	0.697			0.35 [0.17,0.74]	0.006			1.03 [0.46, 2.29]	0.943		
No	0.75 [0.51, 1.12]	0.146			0.45 [0.25, 0.81]	0.008			0.98 [0.49, 1.98]	0.960		
Mode of delivery												
Non-caesarean	1				1				1			
Caesarean	0.87 [0.66, 1.15]	0.325			1.22 [0.76, 1.95]	0.409			0.89 [0.54, 1.46]	0.638		
Diarrhea last two weeks												
No	1				1				1			
Yes	1.11 [0.76, 1.61]	0.598			1.02 [0.54, 1.93]	0.945			0.92 [0.48, 1.79]	0.809		
Fever												
No	1				1				1			
Yes	0.85 [0.66, 1.09]	0.202			0.75 [0.48, 1.20]	0.231			0.69 [0.44, 1.10]	0.119		
Cough												
No	1				1				1			
Yes	1.02 [0.78, 1.32]	0.907			0.68 [0.42, 1.09]	0.107			0.58 [0.35, 0.96]	0.035		
Any infection												
No	1				1				1			
Yes	0.92 [0.72, 1.19]	0.544			0.78 [0.50, 1.21]	0.262			0.75 [0.48, 1.16]	0.193		
Early initiation of breastfeeding												
After 1 h	1				1				1			
Within 1 h	1.64 [1.28, 2.11]	<0.001			1.02 [0.66, 1.60]	0.918			1.16 [0.74, 1.80]	0.521		
Ever breastfeed												
Yes	1				1				1			
No	0.77 [0.58, 1.02]	0.073			1.41 [0.88, 2.27]	0.149			0.85 [0.52, 1.39]	0.510		
Duration of breastfeeding												
Up to 12 months	1				1				1			
>12 months	0.79 [0.55, 1.14]	0.207			0.67 [0.43, 1.04]	0.076			0.42 [0.27, 0.65]	<0.001		

## Data Availability

Data for this research are available online at [https://mics.unicef.org/surveys] (accessed on 16 May 2024).

## Supplementary Materials

The following supporting information can be downloaded at: https://www.mdpi.com/article/10.3390/nu16223893/s1, Table S1. Trends in the prevalence of factors associated with stunting among children (0–23 months) in Tunisia; Table S2. Trends in the prevalence of factors associated with wasting among children (0–23 months) in Tunisia; Table S3. Trends in the prevalence of factors associated with underweight among children (0–23 months) in Tunisia.
