# Supplementary material for: Trends in the Prevalence and Factors Associated with Undernutrition in Tunisian Children Aged 0–23 Months
Source: Nutrients. 2024 Nov 14;16(22):3893. doi: 10.3390/nu16223893 (PMC11597036; doi:10.3390/nu16223893)
Supplement: Supplementary file 1 [file nutrients-16-03893-s001.zip › nutrients-3291263-supplementary.pdf]

Table S1. Trends in the prevalence of factors associated with stunting among children (0-23 months) in Tunisia

Characteristics	2011-2012 Stunting- positive prevalence	2017-18 Stunting-positive prevalence	2022-23 Stunting-positive prevalence	2011-12 - 2017-18	2017-18 - 2022-23	2011-12 - 2022-23
	% (95% CI)	% (95% CI)	% (95% CI)	% (95% CI)	% (95% CI)	% (95% CI)
**Place of residence**						
Urban	9.3 [7.1, 12.2]	9.3 [7.1, 12.2]	16.4 [11.9,22.0]	-2.04 [-5.18, 1.10]	9.08 [3.75, 14.42] ***	7.04 [1.42, 12.66] **
Rural	15.1 [12.0, 18.8]	15.1 [12.0, 18.8]	12.8 [9.1, 17.6]	-4.80 [-9.58, 0.03] **	2.21 [2.81, 7.23]	-2.35[-7.73, 3.03]
**Wealth Index**						
Poorest	8.9 [5.6, 13.8]	10.0 [6.9,14.4]	21.3 [12.1,34.5]	1.12 [-4.37, 6.60]	11.22 [-0.56, 23.00]	12.34 [0.46, 24.22]
Poorer	14.3 [9.9, 20.1]	14.1 [9.5,20.5]	15.5 [9.9,23.6]	-0.13 [7.55, 7.30]	1.41 [-7.28, 10.10]	1.29 [-7.14, 9.71]
Middle	4.6 [2.6, 7.9]	5.2 [3.3,8.2]	12.7 [7.8,19.8]	0.67 [-2.85, 4.19]	7.45 [1.07, 13.82] **	8.12 [1.68, 14.55] **
Fourth	14.1 [9.3, 20.8]	7.0 [4.8,10.2]	9.2 [4.8,17.0]	-7.10 [-13.38, -0.91]	2.16 [-4.27, 8.59]	-4.94 [-13.12, 3.24]
Richest	17.1 [12.8, 22.6]	8.7 [5.5,13.4]	14.4 [9.2,21.9]	-8.41 [-14.64, -2.18]	5.73 [-1.65, 13.11]	-2.68 [-10.64, 5.28]
**Sex of baby**						
Boy	12.9 [10.2,16.1]	8.2 [6.4, 10.5]	16.5 [12.1,22.2]	-4.62 [-8.18, -1.06] **	8.29 [2.89, 13.68] **	3.66 [-2.14, 9.47]
Girl	9.9 [7.4,13.0]	8.9 [6.7, 11.6]	12.8 [9.0,17.7]	-0.98 [-4.68, 2.71]	3.87 [-1.08, 8.82]	2.89 [-2.25, 8.02] NS
**Child age (months)**						
0-5	16.0 [11.9,21.1]	11.3 [8.1,15.5]	27.8 [19.8,37.5]	-4.68 [-10.55, 1.20]	16.48 [6.86, 26, 11] ***	11.81 [1.77, 21.84] **
6-11	5.1 [2.8,9.3]	7.0 [4.5,10.7]	7.8 [4.4,13.5]	1.86 [-2.51, 6.22]	0.85 [-4.47, 6.18]	2.71 [-2.65, 8.08]
12-17	15.5 [11.4,20.8]	5.5 [3.5,8.7]	13.6 [8.0,22.1]	-9.98 [-15.28, -4.68] ***	8.06 [0.67, 15.45] **	-1.92, -10.27, 6.43]
18-23	9.3 [6.3,13.6]	10.3 [7.6,14.0]	10.9 [6.7,17.4]	1.01 [-3.75, 5.77]	0.60 [-5.54, 6.73]	1.61 [-4.74, 7.96]
**Mother's age (years)**						
15-34 years	11.1 [9.0, 13.7]	8.4 [6.7,10.5]	17.2 [13.0, 22.3]	8.82 [3.81, 13.82] ***	6.07 [0.87, 11.27] **	-0.43 [-2.86, 2.01]
35-49 years	12.5 [9.0, 17.1]	8.8 [6.4, 11.9]	11.5 [7.5, 17.2]	2.71 [-2.80, 8.22]	-1.02 [-7.29, 5.24]	-6.99 [-12.66, -1.32] **
**Father's age (years)**						
18-34	10.5 [7.4,14.7]	5.8 [3.8, 8.8]	10.5 [7.4,14.7]	-4.64 [-9.02, -0.26] **	9.52 [2.55, 16.50] **	4.89 [-2.60, 12.37]
35-44	11.5 [8.9,14.9]	9.8 [7.7, 12.6]	11.5 [8.9,14.9]	-1.70 [-5.54, 2.14]	4.51 [-1.00, 10.01]	2.81 [- 2.95, 8.56]
45+	12.0 [7.5,18.5]	8.0 [5.0, 12.5]	12.0 [7.5,18.5]	-3.98 [-10.50, 2.54]	9.28 [0.53, 18.03] **	5.29 [-4.29, 14.88]
**Mother's age at marriage (years)**						
≤ 18	12.6 [5.6,26.1]	6.4 [1.6, 22.7]	12.5 [2.8,41.3]	-6.17 [-19.33, 6.10]	6.07 [-13.39, 25.54]	-0.09 [-20.09, 19.90]
>18	11.4 [9.4,13.7]	8.4 [6.9, 10.1]	15.4 [12.1,19.3]	-2.96 [-5.68, -0.23] **	6.95 [3.01, 10.90] ***	3.99[-0.19, 8.19]
**Marital status**						
Married	11.3 [9.3,13.6]	8.3 [6.8, 10.1]	15.2 [12.0,19.1]	-2.94 [-5.60, -0.28] **	6.87 [2.99, 10.75] ***	3.93 [-6.18, 8.05]
Not married	13.8 [7.8,23.1]	10.8 [6.2, 18.3]	10.8 [3.9,26.7]	-2.95 [-12.49, 6.60]	-0.04 [-12.17, 12.09]	-2.99 [-15.96, 9.99]
**Maternal education**						
Secondary and above	10.1 [7.7, 13.0]	7.9 [6.3,9.8]	13.1 [8.1, 20.5]	-2.15 [-5.30, 0.99]	5.17 [-1.20, 11.55]	3.05 [-3.51, 9.62]
Primary	11.0 [7.9, 15.3]	9.1 [6.0, 13.6]	16.3 [11.9, 21.9]	-1.97 [-7.21, 3.27]	7.20 [0.97, 13.43] **	5.23 [-0.96, 11.42]
No education	20.0 [14.1, 27.4]	13.7 [7.9, 22.6]	16.7 [9.9, 26.7]	-6.30 [-16.09, 3.49]	3.05 [-7.94, 14.05]	-3.25 [-13.87, 7.37]
**Father education**						
Secondary and above	10.1 [7.7, 13.1]	7.7 [6.1, 9.7]	13.1 [8.2, 20.4]	-2.33 [-5.55, 0.89]	5.38 [-0.86, 11.62]	3.05 [-3.51, 9.62]
Primary	10.0 [7.1, 13.9]	9.0 [6.0, 13.4]	16.2 [11.8, 21.9]	-0.10 [-5.96, 3.96]	7.23 [1.00, 13.45] **	6.23 [0.16, 12.30] **
No education	21.6 [15.7, 29.0]	14.8 [9.3, 22.7]	15.4 [9.1, 24.9]	-6.88 [-16.26, 2.51]	0.60 [-9.62, 10.83]	-6.27 [-16.54, 3.99]
**Maternal BMI (kgm^{-2})**						
≤18.5	6.3 [4.7,8.4]	5.9 [4.5, 7.7]	8.5 [5.9,12.0]	-0.43 [-2.86, 2.01]	2.58 [-0.78, 5.95]	2.16 [- 1.35, 5.67]
19-25	19.7 [15.5,24.7]	12.7 [9.8, 16.5]	23.6 [16.9,32.0]	-6.99 [-12.66, -1.32] **	10.88 [2.58, 19.18] **	3.89 [-4.99, 12.76]

25+	32.0 [17.9,50.3]	38.9 [18.8, 63.7]	50.2 [28.1,72.2]	6.96 [-22.34, 36.26]	11.24 [-22.53, 45.02]	18.20 [-10.73, 47.14]
**Household members**						
1-3	9.8 [6.4,14.6]	8.2 [6.2, 10.7]	11.3 [5.7,20.9]	-1.55 [-6.13, 3.03]	3.06 [-4.60, 10.73]	1.51 [-6.84, 9.86]
4-8	12.3 [9.9,15.1]	8.9 [6.9, 11.3]	15.9 [12.3,20.3]	-3.41 [-6.78, -0.04] **	7.03 [2.51, 11.55] **	3.62 [-1.08, 8.32]
>8	10.2 [5.5,18.2]	7.7 [1.0, 40.1]	9.7 [4.0,21.7]	-2.45 [-18.48, 13.58]	1.96 [-15.01, 18.94]	-0.49 [-0.80, 9.83]
**Birth order**						
Non previous	10.4 [7.5,14.2]	8.5 [6.6, 10.8]	18.6 [14.1,24.1]	-1.88 [-5.78, 2.02]	10.13 [4.73, 15.53] ***	8.25 [2.27, 14.23] **
1	11.3 [8.7,14.5]	6.7 [4.5, 9.8]	12.0 [7.4,19.1]	-4.61 [-8.52, -0.69] **	5.37 [-0.93, 11.67]	0.77 [-5.67, 7.20]
2 or more	14.8 [10.1,21.2]	11.9 [8.1, 17.3]	6.6 [2.8,14.9]	-2.86 [-10.03, 4.32]	-5.31 [-12.54, 1.91]	-8.16 [-16.04, -0.30] **
**Source of drinking water**						
Protected	11.6 [9.2, 14.5]	9.3 [7.3, 11.7]	13.9 [10.2, 18.7]	-2.25 [-5.67, 1.18]	4.57 [-0.17, 9.31]	2.33 [-2.64, 7.29]
Unprotected	11.3 [8.5, 15.0]	7.6 [5.7, 10.1]	15.8 [11.1, 21.9]	-3.74 [-7.63, 0.16]	8.18 [2.40, 13.97] **	4.45 [-1.79, 10.69]
**Toilet facility**						
Improved	11.6 [9.2, 14.5]	8.7 [6.5, 11.4]	12.7 [9.1, 17.3]	-2.91 [-6.51, 0.68]	4.02 [-0.68, 8.73]	1.12 [-3.74, 5.96]
Unimproved	11.3[8.6, 14.9]	8.5 [6.6, 10.7]	16.5 [12.0, 22.2]	-2.88 [-6.62, 0.87]	8.03 [2.53, 13.52] **	5.15 [-0.84, 11.14]
**Listening to the radio**						
Not at all	10.6 [8.2,13.7]	8.7 [6.7, 11.1]	15.2 [11.1,20.5]	-1.95 [-5.46, 1.55]	6.55 [1.44, 11.65] **	4.59 [-0.80, 9.98]
Yes	12.2 [9.5,15.5]	8.3 [6.3, 10.9]	14.9 [10.5,20.6]	-3.89 [-7.60, -0.19] **	6.57 [1.07, 12.08] **	2.68 [-3.14, 8.50]
**Watching TV**						
Not at all	20.1 [9.3,38.2]	13.1 [5.1, 29.8]	25.8 [13.4,43.9]	-6.10 [-25.61, 11.62]	12.71 [-6.73, 32.14]	5.71 [-15.42, 26.84]
Yes	11.3 [9.4,13.6]	8.4 [6.9, 10.1]	14.4 [11.3,18.3]	-2.95 [-5.54, -0.36] **	6.06 [2.22, 9.90] **	3.11 [-0.95, 7.17] NS
**Read newspaper**						
Not all	9.0 [6.5,12.4]	8.8 [7.1, 10.7]	15.0 [11.5,19.2]	-2.45 [-3.66, 3.17]	6.20 [1.99, 10.40] **	5.95 [1.16, 10.74] **
Yes	10.9 [8.1,14.5]	7.5 [4.9, 11.2]	15.3 [9.0,24.7]	-3.43 [-7.85, 0.98]	5.61 [-3.07, 14.29] NS	4.39 [-3.98, 12. 76]
**Size of baby**						
Average	11.6 [9.3,14.4]	8.6 [6.8,10.7]	16.2 [12.3,21.0]	-3.05 [-6.25, 0.15]	7.62 [2.87, 12.37] **	4.57 [-0.44, 9.58]
Small	13.7 [8.7,20.8]	15.4 [10.2, 22.5]	14.3 [8.1,24.1]	1.68 [-6.87, 10.22]	-1.05 [-11.01, 8.91]	0.63 [-9.25, 10.51]
Large	8.7 [4.5,16.1]	3.1 [1.5, 6.4]	12.5 [5.7,25.2]	-5.57 [-11.56, 0.41]	9.37 [-0.26, 18.10]	3.80 [-7.08, 14. 68]
**Diarrhoea last two weeks**						
No	11.6 [9.5,13.9]	8.2 [6.7, 10.0]	14.6 [11.4,18.4]	-3.35 [-6.09, -0.61] **	6.35 [2.50, 10.20] ***	3.00 [-1.11, 7.12]
Yes	11.0 [6.5,17.8]	10.9 [6.9, 16.9]	17.4 [7.8,34.4]	-0.05 [-7.45, 7.34]	6.44 [-7.55, 20.43]	6.39 [-7.83, 20.60]
**Fever**						
No	15.5 [10.9,21.6]	8.7 [6.9,10.9]	15.8 [12.2,20.0]	- 6.79 [-12. 48, -1.10] **	7.07 [2.71, 11.44] **	0.28 [- 6.31, 6.87]
Yes	10.4 [8.5,12.8]	11.5 [7.2,18.1]	10.3 [5.9,17.4]	1.10 [-4.66, 6.86]	-1.23 [- 8. 99, 6.52]	-0.13 [-6.15, 5.88]
**Cough**						
No	10.4 [8.2,13.3]	8.5 [6.8, 10.5]	16.1 [12.4,20.7]	-1.95 [-5.09, 1.19]	7.65 [3.15, 12.15] ***	5.70 [0.86, 10.54] **
Yes	12.7 [9.8,16.4]	8.6 [6.1, 12.1]	11 [6.6,17.7]	-4.10 [-8.51, 0.32]	2.35 [-3.84, 8.55]	-1.75 [-8.10, 4.60]
**Any infection**						
No	13.4 [8.2,21.2]	8.8 [6.9, 11.1]	15.3 [11.5,20.1]	-4.63 [-11.33, 2.08]	6.56 [1.85, 11.27] **	1.93 [-5.74, 9.60]
Yes	11.2 [9.3,13.6]	8.2 [6.1, 11.0]	13.6 [9.1,20.0]	-3.00 [-6.23, 0.22]	5.41 [-0.50, 11.32]	2.41 [-3.41, 8.22]
**Place of delivery**						
Government	7.9 [4.9,12.6]	8.5 [7.0, 10.3]	15.3 [11.7,19.8]	0.55 [-3.54, 4.65]	6.84 [2.51, 11.18] **	7.40 [1.90, 12.89] **
Non-government	7.3 [3.6,14.2]	-	15.3 [9.2,24.3]	-7.30 [-12.28, -2.32] **	15.27 [7.78, 22.76] ***	7.96 [-1.04, 16.97]
**Antenatal clinic visits**						

8+	14.3 [10.7,18.8]	8.9 [6.0, 13.0]	19.9 [12.9,29.5]	-5.40 [-10.68, -0.12] **	11.03 [2.09, 19.98] **	5.64 [-3.53, 14.80]
4-7'	9.6 [7.1,12.8]	8.2 [6.3, 10.6]	13.7 [9.7,19.1]	-1.39 [-4.95, 2.17]	5.53 [0.40, 10.67] **	4.15 [-1.32, 9.61] NS
1-3'	11.3 [6.7,18.5]	6.7 [3.8, 11.7]	14.3 [7.8,24.8]	-4.53 [-11.45, 2.39]	7.53 [-1.64, 16.69]	2.10 [-7.1413.14]
None	11.2 [6.7,18.1]	10.8 [7.0, 16.1]	10.1 [5.4,18.1]	-0.42 [-7.59, 6.75]	-0.69 [-8.30, 6.92]	-1.11 [-9.43, 7.20]
**Delivery assistance**						
Skilled	11.0 [8.7,13.8]	8.5[7.0, 10.3]	15.3 [12.1,19.2]	-2.47 [-5.52, 0.58]	6.79 [2.85, 10.72] ***	4.32 [-0.08, 8.72] **
Unskilled	12.4 [9.4,16.2]	8.8 [5.0, 15.0]	8.5 [3.5,19.3]	-3.59 [-9.49, 2.31]	-0.32 [-9.10, 8.47]	-3.91 [-11.96, 4.15]
**Mode of delivery**						
Non-caesarean	11.2 [8.9, 13.9]	9.2 [7.1, 11.7]	17.2 [12.7,22.9]	-2.01 [-5.39, 1.36]	8.06 [2.50, 13.61] **	6.04 [0.40, 11.69] **
Caesarean	11.5 [7.8, 16.7]	7.7 [5.6, 10.4]	13.6 [9.3,19.5]	-3.87 [-8.84, 1.11]	5.91 [0.34, 11.48] **	2.04 [-4.63, 8.72]
**Postnatal checkup**						
0-2 days	14.0 [5.4,31.5]	10.8 [7.0, 6.4]	16.5 [9.7,26.8]	-3.16 [-16.49, 10.18]	5.71 [-3.93, 15.36]	2.56 [-12.54, 17.65]
After 2 days	13.2 [9.6,17.9]	6.9 [3.9, 11.9]	21.6 [11.3,37.4]	-6.32 [-11.96, -0.68] **	14.71 [1.09, 28.33] **	8.39 [-5.32, 11.10]
No	10.6 [8.5,13.2]	8.5 [6.8, 10.5]	13.1 [9.9,17.1]	-2.18 [-5.16, 0.81]	4.62 [0.62, 8.63] **	2.45 [-1.83, 6.72]
**Early initiation of breast feeding**						
After 1 hr	9.4 [7.1,12.2]	8.1 [6.3, 10.2]	10.3 [7.3,14.4]	-1.30 [04.45, 1.85]	2.27 [-1.74, 6.27]	0.97 [-3.35, 5.28]
Withing 1 hr	14.5 [11.4,18.2]	9.4 [7.0, 12.5]	22.2 [16.4,29.4]	-5.09 [-9.43, -0.75] **	12.79 [5.77, 19.81] ***	7.70 [0.38, 15.03] **
**Ever breast feed**						
Yes	13.9 [11.0,17.5]	8.5 [6.9, 10.4]	16 [12.6,20.1]	-5.41 [-9.06, 1.76] **	7.51 [3.38, 11.64] ***	-2.31 [-8.42, 3.79]
No	9.5 [7.2,12.5]	8.8 [5.6, 13.6]	7.2 [3.3,15.1]	-0.74 [-5.43, 3.96]	-1.58 [-8.34, 5.19]	2.09 [-2.87, 7.05]
**Duration of breast feeding (months)**						
Up to 12	13.5 [9.6,18.5]	8.2 [6.1,11.0]	17.9 [13.1,23.9]	2.69 [-2.28, 7.65] NS	9.65 [3.75, 15.55] ***	5.39 [-1.47, 12.25]
>12	10.8 [8.7,13.3]	8.8 [6.9,11.1]	12.5 [8.8,17.4]	-0.57 [-3.74, 2.59]	3.69 [-1.03, 8.41]	2.69 [-2.28, 7.66]
**Literacy**						
Yes	10.2 [6.8,14.9]	8.4 [5.3,13.0]	16.9 [12.5,22.3]	-1 .78 [-7.26, 3.70]	8.45 [2.30, 14.61]	6.67 [0.38, 12.97] **
No	19.3 [14.3,25.6]	8.1 [6.5, 10.0]	13.6 [9.2,19.6]	-11.24 [-17.12, -5.38] ***	5.50 [0.08, 10.91] **	-5.74 [-13.33, 1.84]

- Omitted values
Confidence intervals (CI) = *P< 0.05; **p<0.01; ***p<0.001

Table S2. Trends in the prevalence of factors associated with wasting among children (0-23 months) in Tunisia.

Characteristics	2011-12 Wasting- positive prevalence	2017-18 Wasting- positive prevalence	2022-23 Wasting- positive prevalence	2011-12 & 2017-18	2017-18 & 2022-23	2011-12 & 2022-23
	% (95% CI)	% (95% CI)	% (95% CI)	% (95% CI)	% (95% CI)	% (95% CI)
**Place of residence**						
Urban	2.9 [1.8,4.8]	3.3 [2.1,4.9]	4.8 [2.6,8.6]	0.32 [-1.68, 2.31]	1.52 [-1.63, 4.68]	1.84 [-1.37, 5.05] NS
Rural	5.2 [3.3,8.3]	2.3 [1.3,4.2]	3.6 [2.0,6.3]	-2.90 [-5.72, -0.09]	1.24 [-1.25, 3.74]	-1.66 [-4.86, 1.54] NS
**Wealth Index**						
Poorest	4.0 [2.0,7.8]	2.0 [0.8,4.8]	9.2 [3.8,20.7]	-1.97 [-5.24, 1.30]	7.19 [-0.88, 15.26]	5.22 [-3.12, 13.56]
Poorer	4.6 [2.3,9.0]	3.3 [1.4,7.2]	3.4 [1.5,7.2]	-1.32 [-5.43, 2.80]	0.09 [-3.63, 3.82]	-1.22 [-5.33, 2.88]
Middle	2.3 [0.8,6.2]	3.8 [2.0,7.4]	4.9 [2.4,9.9]	1.56 [-1.87, 4.99]	1.05 [-3.27, 5.38]	2.61 [-1.58, 6.81]
Fourth	5.2 [2.2,11.9]	2.3 [1.1,4.5]	1.7 [0.4,7.0]	-2.98 [-7.67, 1.71]	-0.60 [-3.48, 2.29]	- 3.57 [-8.62, 1.47]
Richest	3.2 [1.8,5.6]	3.8 [1.7,8.2]	2.3 [0.7,7.5]	0.57 [-2.94, 4.08]	-1.41 [-5.50, 2.68]	-0.84 [-4.16, 2.48]

**Sex of baby**						
Boy	4.8 [3.1,7.2]	2.9 [1.8,4.5]	6.3 [3.7,10.3]	-1.91 [-4.29, 0.47]	3.40 [-0.07, 6.87]	1.49 [-2.28, 5.25]
Girl	2.7 [1.4,4.8]	2.9 [1.8,4.9]	2.0 [1.0,4.0]	0.29 [-1.90, 2.49]	-0.98 [-3.03, 1.07]	-0.69 [-2.82, 1.45]
**Child age (months)**						
0-5	6.6 [4.1,10.3]	7.6 [5.0,11.4]	8.2 [4.2,15.4]	1.02 [-3.36, 5.40]	0.59 [-5.64, 6.83]	1.61 [-4.55, 7.78]
6-11	4.4 [2.2,8.8]	2.8 [1.3,5.9]	1.5 [0.5,4.8]	-1.62 [-5.38, 2.14]	-1.24 [-4.00, 1.53]	-2.86 [-6.42, 7.02]
12-17	1.4 [0.4,4.6]	0.7 [0.2,2.4]	1.5 [0.5,4.4]	-0.64 [-2.54, 1.25]	0.81 [-1.03, 2.65]	0.17 [-2.17, 2.51]
18-23	2.3[1.0,5.1]	1.0 [0.3,3.1]	5.5 [2.6,11.3]	-1.34 [-3.53, 0.85]	4.55 [0.32, 8.78] **	3.21[-1.27, 7.68]
**Mother's age (years)**						
15-34 years	3.9 [2.7,5.7]	3.1 [2.1,4.6]	4.2 [2.3,7.6]	-0.87 [-2.80, 1.07]	1.13 [-1.70, 3.96]	0.26 [-2.70, 3.22]
35-49 years	3.4 [1.6, 7.2]	2.6 [1.3,5.0]	4.2 [2.7,6.5]	-0.78 [-3.89, 2.33]	1.60 [-1.48, 4.68]	0.82 [-2.82, 4.47]
**Father's age (years)**						
18-34	4 [2.2,7.4]	4.1 [2.3,7.1]	5.4 [2.2,12.7]	0.06 [-3.31, 3.44]	1.33 [-3.96, 6.61]	1.39 [-3.97, 6.75]
35-44	3.4 [2.0,5.8]	2.5 [1.5,4.2]	3.7 [2.1,6.4]	-0.90 [-3.13, 1.33]	1.16 [-1.29, 3.60]	0.25 [-2.51, 3.02]
45+	3.9 [1.6,9.2]	2.5 [0.9,6.6]	4.3 [1.4,11.9]	-1.35 [-5.58, 2.87]	1.71 [-3.45, 6.88]	0.36 [-5.32, 6.03]
**Mother's age at marriage (years)**						
≤ 18	0.9 [0.1,5.9]	-	-	-0.86 [-2.54, 0.83]	-	-0.86 [-2.55, 0.83]
>18	4 [2.8,5.7]	3.1 [2.2, 4.4]	4.4 [2.8,6.9]	-0.91 [-2.72, 0.89]	1.35 [-0.91, 3.62]	0.44 [-2.02, 2.90]
**Marital status**						
Married	3.7 [2.6,5.3]	3.0 [2.1,4.3]	4.3 [2.7,6.7]	-0.69 [-2.41, 1.03]	1.26 [-0.94, 3.45]	0.57 [-1.78, 2. 92]
Not married	4.7 [1.7,12.1]	1.8 [0.4,7.1]	2.7 [0.4,16.8]	-2.90 [-8.13, 2.33]	0.85 [-4.89, 6.59]	-2.05 [-8.95, 4.85]
**Maternal education**						
Secondary and above	3.8 [2.4,5.9]	2.8 [1.9,4.2]	0.6 [3.0,11.5]	-0.96 [-3.02, 1.10]	3.17 [-1.00, 7.34]	2.21 [-2.14, 6.56]
Primary	3.3 [1.6,6.6]	3.0 [1.5,6.0]	3.4 [1.7,6.9]	-0.28 [-3.43, 2.87]	0.45 [-2.79, 3.68]	0.17 [-3.21, 3.55]
No education	5.2 [2.4,10.8]	4.0 [1.2,12.4]	2.1 [0.5,8.4]	-1.14 [-7.24, 4.97]	-1.95 [-7.51, 3.61]	-3.09 [-8.01, 1.84]
**Father education**						
Secondary and above	4.1 [2.6,6.4]	2.9 [1.9,4.3]	6.3 [3.3, 11.8]	-1.18 [-3.37, 1.02]	3.43 [-0.77, 7.64]	2.26 [-2.18, 6.69]
Primary	2.8 [1.4, 5.5]	2.6 [1.2,5.5]	3.7 [1.9, 7.2]	-0.19 [-2.93, 2.55]	1.09 [-2.10, 4.27]	0.89 [-2.26, 4.05]
No education	4.9 [2.3,10.2]	4.0 [1.4,10.7]	1.2 [0.2,8.4]	-0.87 [-6.35, 4.60]	-2.74 [-7.47, 1.98]	-3.62 [-8.04, 0.80]
**Maternal BMI (kgm-2)**						
≤18.5	5.9 [4.2, 8.3]	4.4 [3.1,6.1]	6.5 [4.2, 10.0]	-1.56 [-4.05, 0.93]	2.19 [-0.97, 5.35]	0.63 [-2.80, 4.07]
19-25	-	-	-	-	-	-
25+	-	-	-	-	-	-
**Household members**						
1-3	6.3 [3.5,11.2]	3.4 [2.2,5.4]	10.2 [4.9,20.2]	-2.90 [-6.90, 1.10]	6.80 [-0.69, 14.30]	3.90 [-4. 30, 12.11]
4-8	2.7 [1.7,4.3]	2.3 [1.4,4.0]	3.0 [1.7,5.2]	-0.38 [-2.14, 1.38]	0.66 [-1.40, 2.72]	0.28 [-1.78, 2.33]
>8	4.9 [1.8,12.4]	7.7 [1.0,40.1]	3.0 [0.4,18.6]	2.85 [-12.69, 18.39]	-4.75 [-20. 66, 11.16]	-1.90 [-9.37, 5.57]
**Birth order**						
Non previous	5.4 [3.3,8.6]	2.8 [1.8,4.5]	5.3 [3.1,9.0]	-2.58 [-5.45, 0.29]	2.51 [-0.60, 5.63]	-0.06 [-3.89, 3.75]
1	3.1 [1.8,5.2]	3.8 [2.1,6.6]	2.3 [1.0,5.5]	0.71 [-1.99, 3.42]	-1.44 [-4.41, 1.52]	-0.73 [-3.35, 1.89]
2 or more	2.1 [0.6,6.9]	1.7 [0.5,5.4]	4.1 [1.2,13.2]	-0.45 [-3.67, 2.77]	2.39 [-2.95, 7.74]	1.94 [-3.63, 7.52]
**Source of drinking water**						
Protected	3.7 [2.3,5.9]	1.7 [0.9,2.9]	4.2 [2.6,6.8]	-2.02 [-4.04, 3.43] **	2.58 [0.34, 4.81] **	0.98 [-2.01, 3.96]
Unprotected	4.0 [2.5,6.2]	4.4 [2.9,6.7]	4.3 [2.0,8.8]	0.42 [-2.15, 2.99]	-0.05 [-3.17, 3.06]	-0.01 [3.22, 3.20]
**Toilet facility**						
Improved	3.7 [2.3,6.1]	1.5 [0.8,3.0]	4.7 [2.8,7.7]	-2.24 [-4.33, -0.16] **	3.22 [0.64, 5.80] **	0.98 [- 2.01, 3.96]

Unimproved	3.9 [2.5,6.0]	3.9 [2.6,5.8]	3.9 [1.9,7.7]	0.05 [-2.26, 2.36]	1.01 [- 1.96, 3.98]	- 0.01 [-3.22, 3.20]
**Listening to the radio**						
Not at all	3.1 [1.8,5.2]	3.1 [2.0,4.9]	4.1 [2.2,7.4]	0.04 [-2.11, 2.19]	0.92 [-1.92, 3.76]	0.96 [-2.00, 3.92]
Yes	4.4 [2.8,6.8]	2.7 [1.6,4.5]	4.4 [2.3,8.2]	-1.70 [-4.12, 0.71]	1.70 [-1.42, 4.82]	0.2 [-3.39, 3.39]
**Watching TV**						
Not at all	-	-	3.8 [0.5,22.5]	-	3.77 [-3.48, 11.04]	-1.03 [-2.79, 0.72]
Yes	3.9 [2.7,5.4]	3.0 [2.1, 4.2]	4.2 [2.7,6.6]	-0.86 [-2.54, 0.81]	0.37 [-1.95, 2.70]	0.14 [-4.37, 4.68]
**Read newspaper**						
Not all	2.0 [1.0,4.3]	3.1 [2.1,4.5]	4.5 [2.8,7.3]	1.06 [-0.86, 2.97]	1.42 [-1.05, 3.90]	2.48 [-0.20, 5.15]
Yes	4.9 [3.1,7.6]	2.3 [0.9,5.4]	2.9 [1.1,7.1]	-2.58 [-5.56, 0.40]	0.59 [-2.72, 3.90]	-1.99 [-5.43, 1.45]
**Size of baby**						
Average	3.7 [2.4,5.8]	2.8 [1.8,4.3]	5.1 [3.1,8.3]	-0.92 [-2.96, 1.12]	2.34 [-0.42, 5.10]	1.42 [-1.57, 4.41]
Small	6.3 [3.1,12.4]	5.1 [2.3,11.3]	3.6 [0.8,14.5]	-1.15 [-7.19, 4.89]	-1.57 [-8.25, 5.10]	-2.72 [-9.54, 4.10]
Large	1.4 [0.4,4.4]	2.5 [1.0,6.3]	1.3 [0.3,6.2]	1.15 [-1.72, 4.02]	-1.24 [-4.38, 1.90]	- 0.09 [-2.73, 2.54]
**Diarrhoea last two weeks**						
No	3.8 [2.6,5.5]	2.8 [1.9,4.0]	4.5 [2.8,6.9]	-1.03 [-2.79, 0.72]	1.68 [-0.54, 3.91]	0.65 [-1.77, 3.07]
Yes	3.7 [1.5,8.7]	3.9 [1.7,8.5]	1.9 [0.3,12.3]	0.14 [-4.37, 4.65]	-1.99 [-6.81, 2.83]	-1.85 [-6.75, 3.05]
**Fever**						
No	4.5 [2.3,8.5]	3.5 [2.5,5.0]	4.4 [2.7,7.0]	-0.91 [-4.12, 2.29]	0.86 [-1.55, 3.27]	-0.05 [-3.66, 3.55]
Yes	3.6 [2.4,5.4]	0.9 [0.2,3.3]	3.5 [1.2,10.4]	-2.77 [-4.63, -0.91] **	2.68 [-1.40, 6.77]	-0.08 [-4.25, 4.08]
**Cough**						
No	4.8 [3.1,7.3]	3.1 [2.1,4.7]	4.3 [2.6,7.1]	-1.64 [-4.04, 0.77]	1.19 [-1.31, 3.68]	-0.45 [-3.43, 2.53]
Yes	2.6 [1.5,4.5]	2.3 [1.2,4.5]	4.0 [1.7,9.4]	-0.26 [-2.37, 1.85]	1.68 [-2.14, 5.51]	1.42 [-2.36, 5.19]
**Any infection**						
No	6.6 [3.0,14.0]	3.3 [2.2,5.1]	4.4 [2.6,7.3]	-3.28 [-8.60, 2.04]	1.04 [-1.64, 3.71]	-2.25 [-7.84, 3.35]
Yes	3.4 [2.3,5.0]	2.3 [1.3,4.0]	4.0 [1.8,8.7]	-1.10 [-2.94, 0.74]	1.64 [-1.78, 5.06]	0.54 [-2.88, 3.96]
**Place of delivery**						
Government	3.9 [1.6,9.1]	3.0 [2.1,4.3]	3.2 [1.9,5.4]	-0.84 [-4.37, 2.70]	0.19 [-1.78, 2.17]	-0.64 [-4.40, 3.1] NS
Non-government	3.7 [1.2,10.5]	0	7.5 [3.3,15.9]	-3.69 [-7.63, 0.26]	7.46 [1.60, 13.33] **	3.78 [-3.30, 10.85]
**Antenatal clinic visits**						
8+	3.2 [1.7,5.8]	4.9 [2.7,8.6]	7.1 [3.2,15.1]	1.68 [-1.75, 5.11]	2.21 [-4.01, 8.44]	3.89 [-1.98, 9.77]
4-7	5.2 [3.3,8.1]	2.0 [1.2,3.5]	3.2 [1.7,5.9]	-3.15 [-5.75, -0.55] **	1.17 [-1.11, 3.43]	-1.98 [-5.04, 1.08]
1-3	0.3 [0.0,2.0]	4.8 [2.1,10.5]	2.3 [0.7,6.8]	4.52 [0.64, 8.41] **	-2.51 [-7.13, 2.11]	2.02 [-0.59, 4.63]
None	3.3 [1.2,8.5]	1.6 [0.5,4.8]	4.7 [1.7,12.4]	-1.70 [-5.37, 1.98]	3.13 [-1.91, 8.17]	1.43 [-4.27, 7.14]
**Delivery assistance**						
Skilled	3.8 [2.5,5.8]	3.1 [2.1,4.3]	4.4 [2.8,6.8]	-0.74 [-2.69, 1.21]	1.30 [-0.93, 3.53]	0.56 [-1.99, 3.12]
Unskilled	3.8 [2.1,6.6]	1.4 [0.3,5.7]	2.9 [0.7,11.1]	-2.36 [-5.29, -.57]	1.49 [-3.02, 5.99]	-0.87 [-5.44, 3.70]
**Mode of delivery**						
Non-caesarean	3.6 [2.4,5.5]	2.6 [1.5,4.3]	4.0 [2.0,7.7]	-1.04 [-3.05, 0.97]	1.40 [1.57, 4.37]	0.36 [-2.69, 3.41]
Caesarean	3.9 [1.7,8.7]	3.6 [2.2,5.8]	4.8 [2.6,8.7]	-0.28 [-3.91, 3.36]	1.14 [-2.25, 4.52]	0.86 [-3.45, 5.17]
**Postnatal checkup**						
0-2 days	13 [4.2,33.7]	5.4 [2.6,10.9]	7.7 [3.3,17.3]	-7.57 [-21.96, 6.83]	2.31 [-5.28, 9.89]	-5 .26[-20.57, 10.05]
After 2 days	2.2 [1.0,4.6]	2.5 [0.9,6.5]	4.3 [1.6,11.4]	0.31 [-2.62, 3.23]	1.83 [-3.12, 6.79]	2.14 [-2.49, 6.76]
No	4.1 [2.7,6.2]	2.5 [1.6,3.8]	3.3 [1.9,5.7]	-1.60 [-3.58, 0.38]	0.76 [-1.33, 2.86]	-0.83 [-3.30, 1.63]
**Early initiation of breast feeding**						
After 1 hr	4.0 [2.6,6.3]	2.6 [1.7,4.1]	4.3 [2.5,7.5]	-1.42 [-3.59, 0.75]	1.70 [-0.98, 4.37]	0.28 [-2.74, 3.29]

Withing 1 hr	3.4 [2.0,5.7]	3.4 [2.0,5.8]	4.1 [2.1,8.0]	-0.02 [-2.57, 2.52]	0.70 [-2.64, 4.05]	0.68 [-2.63, 3.99]
**Ever breast feed**						
Yes	3.1 [1.8,5.1]	3.0 [2.0,4.3]	3.5 [2.2,5.6]	-0.01 [-1.99, 1.79]	0.54 [-1.44, 2.53]	0.44 [-1.82, 2.71]
No	4.4 [2.8,6.8]	2.5 [1.0,6.2]	8.7 [3.3,21.0]	-1.85 [-4.87, 1.18]	6.23 [-2.22, 14.68]	4.38 [-3.99, 12.75]
**Duration of breast feeding**						
Up to 12 months	4.1 [2.2,7.5]	4.9 [3.3,7.3]	3.6 [1.8,7.1]	0.82 [-2.39, 4.02]	-1.30 [-4.48, 1.87]	-0.49 [-4.0, 3.03]
>12 months	3.7 [2.4,5.5]	1.4 [0.8,2.7]	4.7 [2.7,8.2]	-2.25 [-4.01, -0.49]	3.28 [0.51, 6.06] **	1.03 [-1.99, 4.07]
**Literacy**						
Yes	3.1 [1.4,7.1]	1.4 [0.4,4.5]	3.8 [1.9,7.4]	-1.78 [-4.85, 1.29]	2.44 [-0.58, 5.47]	0.66 [-2.97, 4.30]
No	4.7 [2.4,9.1]	3.2 [2.2,4.6]	4.4 [2.3,8.2]	-1.51 [-4.89, 1.87]	1.21 [-1.83, 4.25]	-0.30 [-4.53, 3.93]

- Omitted values

Confidence intervals (CI) = *P< 0.05; **p<0.01; ***p<0.001

Table S3. Trends in the prevalence of factors associated with underweight among children (0-23 months) in Tunisia

Characteristics	2011-12 underweight-positive prevalence	2017-18 underweight-positive prevalence	2022-23 underweight-positive prevalence	2011-12 & 2017-18	2017-18 & 2022-23	2011-12 & 2022-23
	% (95% CI)	% (95% CI)	% (95% CI)	% (95% CI)	% (95% CI)	% (95% CI)
**Place of residence**						
Urban	2.9 [1.8,4.6]	2.5 [1.6,3.9]	6.2 [3.5,10.8]	-0.39 [-2.13, 1.35]	3.66 [-0.05, 7.36] **	3.27 [-0.52, 7.05]
Rural	4.2 [2.4,7.1]	2.5 [1.4,4.6]	4.7 [2.8,7.6]	-1.65 [-4.36, 1.06]	2.16 [-0.60, 4.91]	0.51 [-2.70, 3.72]
**Wealth Index**						
Poorest	3.8 [1.9,7.4]	3.2 [1.5,6.6]	12.1 [5.6,24.2]	-0.52 [-4.04, 2.10]	8.86 [-0.37, 18. 08]	8.34 [-0.95, 17.63]
Poorer	3.2 [1.5,6.8]	4.0 [2.0,7.8]	4.3 [2.2,8.2]	0.78 [-2.87, 4.43]	0.28 [-3.64, 4.20]	1.06 [-2.70, 4.82]
Middle	2.2 [0.7,6.8]	1.9 [0.7,5.1]	7.2 [3.3,15.3]	-0.32 [-3.48, 2.85]	5.36 [-0.56, 11.29]	5.05 [-1.11, 11.20]
Fourth	2.6 [0.9,6.9]	2.1 [1.0,4.3]	1.6 [0.5,5.1]	-0.52 [-3.50, 2.47]	-0.41 [-2.81, 1.10]	-0.92 [-4.12, 2.26]
Richest	4.8 [2.7,8.5]	2.1 [0.9,4.9]	2.3 [0.9,6.3]	-2.72 [-6.02, 0.59]	0.25 [-2.69, 3.19]	-2.47 [-6.10, 1.17]
**Sex of baby**						
Boy	4.7 [3.1,7.1]	3.0 [1.9,4.6]	7.5 [4.7,11.7]	-1.72 [-4.05, 0.61]	4.49 [0.86, 8.13] **	2.77 [-1.13, 6.68] NS
Girl	1.8 [0.9,3.6]	2.0 [1.1,3.6]	3.2 [1.4,7.3]	0.13 [-1.60, 1.85]	1.28 [-1.65, 4.21]	1.41 [-1.55, 4.37] NS
**Child age**						
0 to 5	6.9 [4.3,10.8]	7.6 [5.0,11.4]	15.7 [9.4,25.1]	0.74 [-3.72, 5.21]	8.15 [-0.21, 16.50]	8.89 [0.52, 17.26] **
6 to 11	2.6 [1.1,5.8]	1.0 [0.4,2.8]	4.6 [2.2,9.3]	-1.54 [-3.91, 0.82]	3.52 [0.07, 6.97] **	1.98 [-1.95, 5.90]
12 to17	0.7 [0.2,2.3]	0.1 [0.0,0.9]	2.3 [0.6,9.3]	-0.53 [-1.40, 0.34]	2.20 [-1.12, 5.52]	1.67 [-1.74, 5.08]
18 to 23	3.0 [1.4,6.5]	1.7 [0.7,4.1]	1.2 [0.4,3.8]	-1.30 [-4.09, 1.50]	-0.46 [-2.52, 1.59]	-1.76 [-4.51, 0.99]
**Mother's age**						
15-34 years	3.7 [2.5,5.5]	2.5 [1.6,3.9]	6.6 [4.0,10.6]	-1.21 [-3.02, 0.61]	4.07 [0.69, 7.44] **	2.86 [-0.64, 6.36]
35-49 years	2.5 [1.1, 5.5]	2.6 [1.4,4.7]	3.7 [1.8, 7.5]	0.16 [-2.40, 2.72]	1.07 [-2.02, 4.16]	1.23 [-2.11, 4.58]
**Father's age**						
18-34	6.4 [3.9,10.3]	3.1 [1.7,5.8]	5.7 [2.5,12.3]	-3.27 [-6.93, 0.38]	2.61 [-2.32, 7.54]	-0.66 [-6.15, 4.83] NS
35-44	2.0 [1.1,3.6]	2.1 [1.2,3.6]	5.2 [2.9,9.1]	0.08 [-1.55, 1.71]	3.12 [-0.06, 6.30] **	3.20 [0.013, 6.38] **
45+	3.1 [1.1,8.6]	2.7 [1.1,6.5]	6.5 [2.6,15.5]	-0.40 [-4.44, 3.64]	3.79 [-2.56, 10.14]	3.39 [-3.32, 10.10] NS
**Mother age at marriage**						
≤ 18 years	-	-	-	-	9.17 [-7.88, 26.22]	9.17 [-7.88, 26.22]
>18 years	3.7 [2.6,5.3]	2.6 [1.8,3.7]	5.4 [3.5,8.2]	-1.16 [-2.80, 0.47]	2.87 [0.39, 5.34] **	1.70 [-0.94, 4.35]
**Marital status**						
Married	3.6 [2.5,5.2]	2.5 [1.7,3.6]	5.6 [3.7,8.4]	-1.12 [-2.72, 0.48]	3.10 [0.61, 5.60] **	1.98 [-0.67, 4.64] NS
Not married	1.0 [0.1,7.1]	3.1 [1.1,8.6]	2.5 [0.3,15.9]	2.04 [-1.78, 5.87]	-0.59 [-6.42, 5.24]	1.45 [-3.81, 6.71] NS
**Maternal education**						
Secondary and above	3.9 [2.5,6.1]	2.4 [1.6,3.7]	5.8 [2.7,11.9]	-1.52 [-3.53, 0.49]	3.38 [-1.05, 7.81]	1.86 [-2.79, 6.51]
Primary	1.6 [0.7,3.4]	2.9 [1.4,5.9]	4.0 [2.1,7.2]	1.29 [-1.15, 3.72]	1.08 [-2.13, 4.28]	2.36 [-0.34, 5.06]
No education	4.9 [2.3,10.4]	3.2 [0.9,11.1]	11.4 [5.1,23.7]	-1.76 [-7.33, 3.81]	8.22 [-1.57, 18.02]	6.46 [-3.21, 16.13]
**Father education**						
Secondary and above	3.9 [2.5,6.1]	2.3 [1.5,3.6]	5.4 [2.5,11.3]	-1.55 [-3.59, 0.50	3.01 [-1.21, 7.24]	1.47 [2.99, 5.93] NS
Primary	1.5 [0.7,3.3]	2.5 [1.1,5.4]	4.7[2.7, 8.1]	0.98 [-1.30, 3.26]	2.17 [-1.10, 5.44]	3.15 [0.28, 6.01] **
No education	5.4 [2.6,10.7]	4.3 [1.8,10.3]	10.0 [4.2,21.9]	-1.04 [-6.45, 4.37]	5.64 [-3.52, 14.80]	4.61 [-4.53, 13.74]
**Maternal BMI**						
≤18.5	4.6 [3.1,6.7]	3.6 [2.5,5.1]	6.6 [4.0,10.5]	-1.01 [-3.20, 1.17]	2.99 [-0.42, 6.40]	1.98 [-1.62, 5.57]

19-25	1.0 **[0.3,3.2]**	0.5 [0.2,1.2]	3.9 [1.9,7.9]	-0.56 [-1.83, 0.71]	3.43 [0.60, 6.25] **	2.87 [-0.16, 5.89]
25+	2.4 [0.3,15.4]	-	-	-2.41 [-7.11, 2.29]	-	-2.41 [-7.12, 2.29]
**Household members**						
1-3	4.5 [2.3,8.6]	2.2 [1.3,3.8]	3.5 [1.2,9.5]	-2.22 [-5.44, 0.99]	1.26 [-2.53, 5.05]	-0.96 [-5.63, 3.70]
4-8	3.3 [2.2,5.0]	2.7 [1.7,4.4]	6.0 [3.8,9.3]	-0.56 [-2.46, 1.35]	3.23 [0.24, 6.21] **	2.67 [-0.36, 5.70]
>8	1.0 [0.3,3.5]	7.7 [1.0,40.1]	5.5 [1.6,17.2]	6.72 [-8.15, 21.59]	-2.26 [-18.48, 13.95]	4.46 [-2.27, 11.18]
**Birth order**						
Non previous	3.3 [1.8,6.0]	2.5 [1.6,4.0]	7.4 [4.7,11.6]	-0.78 [-3.11, 1.54]	4.89 [1.34, 8.44] **	4.10 [0.20, 8.01] **
1	3.3 [1.9,5.5]	1.3 [0.5,3.3]	2.0 [0.5,8.0]	-1.97 [-4.08, 0.15]	0.66 [-2.42, 3.74]	-1.31 [-4.61, 2.00]
2 or more	3.9 [1.8,8.1]	4.6 [2.4,8.8]	5.6 [2.0,14.7]	0.71 [-3.47, 4.89]	0.98 [-5.41, 7.38]	1.69 [-4.64, 8.02]
**Source of drinking water**						
Protected	4.0 [2.6,6.1]	1.8 [1.1,3.0]	5.3 [3.3,8.4]	-2.19 [-4.14, -0.25] **	3.51 [0.87, 6.14] **	1.31 [-1.67, 4.30] NS
Unprotected	2.4 [1.3,4.4]	3.4 [2.1,5.4]	5.7 [2.9,10.9]	1.01 [-1.15, 3.17]	2.32 [-1.78, 6.42]	3.33 [-0.72, 7.38] NS
**Toilet facility**						
Improved	4.0 [2.6,6.1]	1.4 [0.7,2.6]	4.0 [2.3,6.8]	-2.63 [-4.57, -0.69] **	2.58 [0.22, 4.94] **	-0.05 [-2.83, 2.74]
Unimproved	2.4 [1.3,4.4]	3.4 [2.2,5.1]	6.8 [4.0,11.3]	0.94 [-1.07, 2.93]	3.39 [-0.42, 7.20]	4.33 [0.50, 8.15] **
**Listening to the radio**						
Not at all	2.4 [1.4,4.0]	2.4 [1.5,3.9]	5.4 [3.2,9.0]	0.01 [-1.67, 1.70]	3.02 [0.79, 6.04] **	3.04 [-0.02, 6.09] **
Yes	4.2 [2.7,6.6]	2.7 [1.6,4.6]	5.6 [3.0,10.3]	-1.49 [-3.87, 0.89]	2.83 [-0.92, 6.58]	1.34 [-2.62, 5.30]
**Watching TV**						
Not at all	2.9 [0.4,18.1]	9.4 [2.4,30.9]	8.4 [2.5,24.6]	6.53 [-7.11, 20.17]	-0.96 [-16.76, 14.84]	5.57 [-5.68, 16.82] NS
Yes	3.4 [2.4,4.8]	2.4 [1.7,3.4]	5.3 [3.4,8.1]	-1.01 [-2.50, 0.47]	2.95 [0.50, 5.40] **	1.93 [-0.66, 4.52] NS
**Read newspaper**						
Not all	1.7 [0.8,3.8]	2.8 [1.9,4.0]	6.1 [4.0,9.4]	1.05 [-0.66, 2.76]	3.37 [0.51, 6.23] **	4.42 [1.44, 7.41] **
Yes	4.2 [2.6,6.8]	1.7 [0.6,4.5]	2.6 [0.9,6.9]	-2.54 [-5.15, 0.07]	0.88 [-2.19, 3.96]	-1.66 [-4.93, 1.62]
**Size of baby**						
Average	3.6 [2.4,5.3]	2.3 [1.4,3.7]	5.1 [3.0,8.7]	-1.26 [-3.07, 0.54]	2.83 [-0.15, 5.81]	1.57 [-1.57, 4.70]
Small	7.0 [3.2,14.6]	5.4 [2.6,10.8]	5.6 [1.9,15.1]	-1.65 [-8.21, 4.90]	0.20 [-6.76, 7.16]	-1.45 [-9.33, 6.43]
Large	0.5 [0.1,2.1]	1.4 [0.4,4.7]	7.8 [3.5,16.6]	0.86 [-1.02, 2.74]	6.44 [0.07, 12.81] **	7.30 [1.12, 13.48] **
**Diarrhoea last two weeks**						
No	3.5 [2.4,5.0]	2.5 [1.7,3.6]	5.6 [3.7,8.5]	-0.10 [-2.60, 0.61]	3.15 [0.63, 5.66] **	2.15 [-0.52, 4.82] NS
Yes	2.9 [1.1,7.4]	3.1 [1.2,7.5]	4.8 [1.2,17.8]	0.17 [-3.76, 4.12]	1.77 [-5.46, 9.00]	1.94 [-5.28, 9.16] NS
**Fever**						
No	3.3 [1.5,7.5]	3.0 [2.1,4.3]	6.1 [4.0,9.3]	-0.34 [-3.28, 2.60]	3.14 [0.34, 5.93] **	2.80 [-0.95, 6.55]
Yes	3.4 [2.3,5.0]	1.0 [0.2,3.8]	2.5 [0.7,9.2]	-2.42 [-4.31, -0.53] **	1.56 [-2.06, 5.19]	-0.85'[-4.46, 2.76]
**Cough**						
No	4.5 [3.0,6.8]	2.7 [1.8,4.1]	5.9 [3.7,9.2]	-1.82 [-4.00, 0.36]	3.19 [0.31, 6.07] **	1.37 [-1.89, 4.63] NS
Yes	2.0 [1.0,3.8]	2.1 [1.0,4.3]	4.4 [1.8,10.2]	0.09 [-1.91, 2.09]	2.29 [-1.81, 6.39]	2.38 [-1.65, 6.40] NS
**Any infection**						
No	5.3 [2.1,12.7]	2.7 [1.7,4.2]	6.0 [3.7,9.6]	-2.63 [-7.56, 2.29]	3.33 [0.24, 6.43] **	0.70 [-4.87, 6.27]
Yes	3.1 [2.1,4.6]	2.3 [1.3,4.1]	4.4 [2.1,9.2]	-0.81 [-2.59, 0.97]	2.12 [-1.42, 5.67]	1.31 [-2.18, 4.81]
**Place of delivery**						
Government	3.0 [1.3,7.2]	2.5 [1.7,3.7]	4.4 [2.7,7.0]	-0.52 [-3.35, 2.31]	1.82 [-0.47, 4.10]	1.30 [-2.08, 4.69]
Non-government	4.1 [1.6,9.9]	0	9.5 [4.5,18.9]	-4.11 [-7.81, -0.41] **	9.53 [2.69, 16.38] **	5.42 [-2.37, 13.21]
**Antenatal clinic visits**						
8+	4.0 [2.3,6.9]	1.5 [0.6,4.0]	3.9 [1.5,9.6]	-2.50 [-5.16, 0.16]	2.31 [-1.59, 6.21]	-0.19 [-4.43, 4.06]

4-7	4.0 [2.4,6.7]	2.3 [1.4,3.8]	6.8 [3.9,11.7]	-1.72 [-4.07, 0.64]	4.51 [0.60, 8.42] **	2.79 [-1.46, 7.05]
1-3	1.4 [0.4,5.0]	4.3 [1.9,9.7]	3.9 [1.5,9.8]	2.89 [-1.10, 6.88]	-0.45 [-5.56, 4.67]	2.45 [-1.64, 6.53]
None	1.1 [0.3,4.3]	3.6 [1.6,8.0]	5.3 [2.1,13.0]	2.52 [-0.78, 5.82]	1.71 [-4.01, 7.43]	4.23 [-0.91, 9.37]
**Delivery assistance**						
Skilled	3.8 [2.5,5.8]	2.5 [1.7,3.7]	5.7 [3.8,8.6]	-1.27 [-3.13, 0.60]	3.18 [0.64, 5.73] **	1.92 [-o.94, 4.77] NS
Unskilled	2.6 [1.4,4.9]	2.4 [0.8,6.9]	2.9 [0.7,11.2]	-0.17 [-3.21, 2.88]	0.49 [-4.32, 5.29]	0.32 [-4.06, 4.70] NS
**Mode of delivery**						
Non-caesarean	3.7 [2.4,5.5]	2.9 [1.8,4.6]	5.7 [3.3,9.6]	-0.77 [-2.80, 1.27]	2.79 [-5.31, 6.12]	2.03 [-1.37, 5.42]
Caesarean	3.4 [1.5,7.3]	2.1 [1.1,3.9]	5.8 [3.0,10.8]	-1.31 [-4.27, 1.66]	3.67 [-2.36, 7.58]	2.36 [-2.19, 6.91]
**Postnatal checkup**						
0-2 days	9.5 [2.5,30.0]	4.0 [1.9,8.5]	0.9 [0.2,3.7]	-5.43 [-17.91, 7.05]	-3.14 [-6.47, 0.19]	-8.57 [-20.74, 3.59]
After 2 days	3.4 [1.7,6.5]	2.1 [0.8,5.4]	8.1 [3.4,18.4]	-1.24 [-4.24, 1.76]	5.99 [-1.26, 13.25]	4.75 [-2.56, 12.07]
No	3.1 [2.0,4.9]	2.3 [1.5,3.6]	6.3 [3.9,9.8]	-0.81 [-2.53, 0.91]	3.92 [0.86, 6.97] **	3.12 [-0.09, 6.30]
**Early initiation of breast feeding**						
After 1 hr	2.9 [1.7,4.9]	2.4 [1.5,3.7]	5.8 [3.5,9.4]	-0.55 [-2.42, 1.31]	3.40 [0.32, 6.48] **	2.85 [-0.43, 6.12] NS
Withing 1 hr	4.0 [2.5,6.4]	2.8 [1.6,5.0]	5.0 [2.6, 9.7]	-1.21 [3.70, 1.27]	2.24 [-1.48, 5.96]	1.02 [-2.82, 4.86] NS
**Ever breast feed**						
Yes	3.8 [2.3, 6.0]	3.8 [2.4,5.9]	6.0 [3.9, 9.0]	-1.35 [-3.36, 0.67]	3.55 [0.87, 6.23] **	2.20 [-0.86, 5.27] NS
No	3.1 [1.8, 5.2]	1.6 [0.9,2.9]	2.7 [0.8, 8.1]	0.12 [-2.67, 2.88]	-0.52 [-4.32, 3.28]	-0.42 [-3.86, 3.03] NS
**Duration of breast feeding**						
Up to 12 months	4.7 [2.78.0]	3.1 [1.1, 8.0]	9.7 [6.1, 15.2]	1.72 [-1.15, 4.60]	7.39 [2.60, 12.18] **	7.39 [2.60, 12.18] **
>12 months	2.9 [1.9, 4.6]	3.3 [2.3, 4.6]	2.3 [1.1, 5.0]	2.13 [0.20, 4.06] **	2.13 [0.20, 4.06] **	1.72 [-1.16, 4.60]
**Literacy**						
Yes	1.2 [0.5,3.1]	1.3 [0.4,3.8]	6.6 [4.0,10.9]	5.38 [1.74, 9.03] **	5.45 [1.90, 9.00] **	5.45 [1.90, 9.00] **
No	4.9 [2.5,9.2]	2.8 [1.9,4.1]	4.4 [2.2,8.7]	1.62 [-1.64, 4.88]	-0.46 [-4.90, 3.97]	-0.46 [-4.90, 3.97]

- Omitted values

Confidence intervals (CI) = *P< 0.05; **p<0.01; ***p<0.001
